# Supplementary material for: MeSH-Informed Enrichment Analysis and MeSH-Guided Semantic Similarity Among Functional Terms and Gene Products in Chicken
Source: G3 (Bethesda). 2016 Jun 2;6(8):2447–53. doi: 10.1534/g3.116.031096 (PMC4978898; doi:10.1534/g3.116.031096)
Supplement: Supplemental Material [file supp_6_8_2447__index.html]

MeSH-Informed Enrichment Analysis and MeSH-Guided Semantic Similarity Among Functional Terms and Gene Products in Chicken — Supplemental Material 

# MeSH-Informed Enrichment Analysis and MeSH-Guided Semantic Similarity Among Functional Terms and Gene Products in Chicken

## Supplemental Material for Morota, Beissinger, and Penagaricano *et al*, 2016

**Files in this Data Supplement:**

- File S1 - MeSH over-representation analysis (RNA-seq data). (.zip, 2.73 MB)
- File S2 - MeSH over-representation analysis (Selective sweep data). (.zip, 2.5 MB)
- File S3 - Gene Semantic Similarity (RNA-seq data). (.zip, 3.44 MB)
- File S4 - Gene Semantic Similarity (Selective sweep data). (.zip, 3.24 MB)
