# Supplementary material for: MeSH-Informed Enrichment Analysis and MeSH-Guided Semantic Similarity Among Functional Terms and Gene Products in Chicken
Source: G3 (Bethesda). 2016 Jun 2;6(8):2447–53. doi: 10.1534/g3.116.031096 (PMC4978898; doi:10.1534/g3.116.031096)
Supplement: Supplemental Material [file supp_g3.116.031096_FileS1.zip › FileS1.html]

MeSH over-representation analysis (RNA-seq data)


# MeSH over-representation analysis (RNA-seq data)

## 1. Create a vector of background genes

We first create a vector of background genes.

```
library(biomaRt)
## access to biomaRt
#mart <- useMart(biomart = "ensembl", dataset = "ggallus_gene_ensembl",host = "www.ensembl.org")
mart <- useMart(biomart = "ENSEMBL_MART_ENSEMBL", dataset = "ggallus_gene_ensembl", 
                host = "www.ensembl.org")
univ.geneID <- getBM(attributes=c("ensembl_gene_id", "entrezgene", 
                                  "hgnc_symbol"), mart = mart) # 17680 
## remove genes with no corresponding Entrez Gene ID
univ.geneID2 <- univ.geneID[!is.na(univ.geneID[,2]),] # 14124 
## remove duplicated Entrez Gene ID
univ.geneID3 <- univ.geneID2[ !duplicated(univ.geneID2[,2]),] # 13984
```

# 2. Create a vector of selected genes

Secondly, we create a vector of significant genes by reading the input file.

```
## read data
my.geneID <- read.table("rnaseqGenes.txt", header=FALSE) # 286
colnames(my.geneID) <- "ensembl_gene_id"
## merge two files
my.geneID2 <- merge(my.geneID, univ.geneID3, by ="ensembl_gene_id") # 263
## remove duplicated Entrez Gene ID
my.geneID3 <- my.geneID2[ !duplicated(my.geneID2$entrezgene),] # 263
```

## 3. GO enrichment analysis

We perform a GO analysis using the *GOstats* package.

```
library("org.Gg.eg.db")
library("GOstats")
library("GOSemSim")
paraGO <- new("GOHyperGParams", geneIds=my.geneID3[,2], universeGeneIds=univ.geneID3[,2], 
              annotation="org.Gg.eg.db", ontology="BP", pvalueCutoff=0.05, 
              conditional=TRUE, testDirection="over")
```

GO enrichment analysis for **BP**

```
BP <- hyperGTest(paraGO)
summary(BP)[,c(1,2,7)] # 136
```

```
##         GOBPID       Pvalue
## 1   GO:0006066 0.0001346985
## 2   GO:0034433 0.0023178686
## 3   GO:0034435 0.0023178686
## 4   GO:0072273 0.0023178686
## 5   GO:0090190 0.0023178686
## 6   GO:0008203 0.0039322704
## 7   GO:0031640 0.0045517534
## 8   GO:0046470 0.0045517534
## 9   GO:0061217 0.0045517534
## 10  GO:0072077 0.0045517534
## 11  GO:0045445 0.0050105534
## 12  GO:0090090 0.0062507020
## 13  GO:0006639 0.0074490804
## 14  GO:0043534 0.0074490804
## 15  GO:0050832 0.0074490804
## 16  GO:0090184 0.0074490804
## 17  GO:0042572 0.0109719667
## 18  GO:0051057 0.0109719667
## 19  GO:0051496 0.0109719667
## 20  GO:0030198 0.0109979825
## 21  GO:0048588 0.0109979825
## 22  GO:0042445 0.0129371294
## 23  GO:0008202 0.0135341138
## 24  GO:0032501 0.0135775266
## 25  GO:0030111 0.0141272414
## 26  GO:0007399 0.0144044785
## 27  GO:0001838 0.0150840702
## 28  GO:0016126 0.0150840702
## 29  GO:0032231 0.0150840702
## 30  GO:0032331 0.0150840702
## 31  GO:0048771 0.0150840702
## 32  GO:0070271 0.0164713600
## 33  GO:0018108 0.0165179070
## 34  GO:0016101 0.0197505358
## 35  GO:0070167 0.0197505358
## 36  GO:0007015 0.0225843317
## 37  GO:0006576 0.0249379427
## 38  GO:0030168 0.0249379427
## 39  GO:0035148 0.0249379427
## 40  GO:0051651 0.0249379427
## 41  GO:0007169 0.0251089934
## 42  GO:0010631 0.0254228285
## 43  GO:0071822 0.0271832002
## 44  GO:0030502 0.0277777778
## 45  GO:0032488 0.0277777778
## 46  GO:1903011 0.0277777778
## 47  GO:0001907 0.0283286119
## 48  GO:0003257 0.0283286119
## 49  GO:0003337 0.0283286119
## 50  GO:0006000 0.0283286119
## 51  GO:0006116 0.0283286119
## 52  GO:0006930 0.0283286119
## 53  GO:0007538 0.0283286119
## 54  GO:0010804 0.0283286119
## 55  GO:0010898 0.0283286119
## 56  GO:0010903 0.0283286119
## 57  GO:0014029 0.0283286119
## 58  GO:0014036 0.0283286119
## 59  GO:0018158 0.0283286119
## 60  GO:0018206 0.0283286119
## 61  GO:0019100 0.0283286119
## 62  GO:0019217 0.0283286119
## 63  GO:0019432 0.0283286119
## 64  GO:0019836 0.0283286119
## 65  GO:0021800 0.0283286119
## 66  GO:0030300 0.0283286119
## 67  GO:0032371 0.0283286119
## 68  GO:0032489 0.0283286119
## 69  GO:0032692 0.0283286119
## 70  GO:0033344 0.0283286119
## 71  GO:0033564 0.0283286119
## 72  GO:0033700 0.0283286119
## 73  GO:0034115 0.0283286119
## 74  GO:0034380 0.0283286119
## 75  GO:0035025 0.0283286119
## 76  GO:0035382 0.0283286119
## 77  GO:0035622 0.0283286119
## 78  GO:0036032 0.0283286119
## 79  GO:0042632 0.0283286119
## 80  GO:0044058 0.0283286119
## 81  GO:0044179 0.0283286119
## 82  GO:0044241 0.0283286119
## 83  GO:0045723 0.0283286119
## 84  GO:0046460 0.0283286119
## 85  GO:0046851 0.0283286119
## 86  GO:0050713 0.0283286119
## 87  GO:0050994 0.0283286119
## 88  GO:0051006 0.0283286119
## 89  GO:0051457 0.0283286119
## 90  GO:0051659 0.0283286119
## 91  GO:0051801 0.0283286119
## 92  GO:0051818 0.0283286119
## 93  GO:0051973 0.0283286119
## 94  GO:0055091 0.0283286119
## 95  GO:0060009 0.0283286119
## 96  GO:0060221 0.0283286119
## 97  GO:0060354 0.0283286119
## 98  GO:0060517 0.0283286119
## 99  GO:0060532 0.0283286119
## 100 GO:0060534 0.0283286119
## 101 GO:0060729 0.0283286119
## 102 GO:0060761 0.0283286119
## 103 GO:0060784 0.0283286119
## 104 GO:0061046 0.0283286119
## 105 GO:0061145 0.0283286119
## 106 GO:0065005 0.0283286119
## 107 GO:0070327 0.0283286119
## 108 GO:0070384 0.0283286119
## 109 GO:0070508 0.0283286119
## 110 GO:0071504 0.0283286119
## 111 GO:0072190 0.0283286119
## 112 GO:0072193 0.0283286119
## 113 GO:0072197 0.0283286119
## 114 GO:0072289 0.0283286119
## 115 GO:0090037 0.0283286119
## 116 GO:0090191 0.0283286119
## 117 GO:0090207 0.0283286119
## 118 GO:0090291 0.0283286119
## 119 GO:1900086 0.0283286119
## 120 GO:1900155 0.0283286119
## 121 GO:1900158 0.0283286119
## 122 GO:1902027 0.0283286119
## 123 GO:2000020 0.0283286119
## 124 GO:2000138 0.0283286119
## 125 GO:2000741 0.0283286119
## 126 GO:2001054 0.0283286119
## 127 GO:0001656 0.0306142539
## 128 GO:0006720 0.0306142539
## 129 GO:0035821 0.0306142539
## 130 GO:0043623 0.0322004563
## 131 GO:0009605 0.0332254655
## 132 GO:0051707 0.0341520596
## 133 GO:0090130 0.0351728486
## 134 GO:0007155 0.0354251530
## 135 GO:0001525 0.0361338416
## 136 GO:0009308 0.0367487668
## 137 GO:0050829 0.0367487668
## 138 GO:0060675 0.0367487668
## 139 GO:0061008 0.0367487668
## 140 GO:0072078 0.0367487668
## 141 GO:0009607 0.0382746520
## 142 GO:0006644 0.0387962052
## 143 GO:2000147 0.0426036065
## 144 GO:0016055 0.0431949255
## 145 GO:0046474 0.0433120661
## 146 GO:0060993 0.0433120661
## 147 GO:0061326 0.0433120661
## 148 GO:0072330 0.0433120661
## 149 GO:0022607 0.0450528552
## 150 GO:0007275 0.0496555223
##                                                                                                                           Term
## 1                                                                                                    alcohol metabolic process
## 2                                                                                                       steroid esterification
## 3                                                                                                   cholesterol esterification
## 4                                                                                            metanephric nephron morphogenesis
## 5                                                      positive regulation of branching involved in ureteric bud morphogenesis
## 6                                                                                                cholesterol metabolic process
## 7                                                                                           killing of cells of other organism
## 8                                                                                        phosphatidylcholine metabolic process
## 9                                                                                        regulation of mesonephros development
## 10                                                                                                 renal vesicle morphogenesis
## 11                                                                                                    myoblast differentiation
## 12                                                                      negative regulation of canonical Wnt signaling pathway
## 13                                                                                              acylglycerol metabolic process
## 14                                                                                     blood vessel endothelial cell migration
## 15                                                                                                  defense response to fungus
## 16                                                                                   positive regulation of kidney development
## 17                                                                                                   retinol metabolic process
## 18                                                            positive regulation of small GTPase mediated signal transduction
## 19                                                                                positive regulation of stress fiber assembly
## 20                                                                                           extracellular matrix organization
## 21                                                                                                   developmental cell growth
## 22                                                                                                   hormone metabolic process
## 23                                                                                                   steroid metabolic process
## 24                                                                                            multicellular organismal process
## 25                                                                                         regulation of Wnt signaling pathway
## 26                                                                                                  nervous system development
## 27                                                                                         embryonic epithelial tube formation
## 28                                                                                                 sterol biosynthetic process
## 29                                                                                regulation of actin filament bundle assembly
## 30                                                                          negative regulation of chondrocyte differentiation
## 31                                                                                                           tissue remodeling
## 32                                                                                                  protein complex biogenesis
## 33                                                                                           peptidyl-tyrosine phosphorylation
## 34                                                                                               diterpenoid metabolic process
## 35                                                                                 regulation of biomineral tissue development
## 36                                                                                                 actin filament organization
## 37                                                                                   cellular biogenic amine metabolic process
## 38                                                                                                         platelet activation
## 39                                                                                                              tube formation
## 40                                                                                             maintenance of location in cell
## 41                                                            transmembrane receptor protein tyrosine kinase signaling pathway
## 42                                                                                                   epithelial cell migration
## 43                                                                                        protein complex subunit organization
## 44                                                                                  negative regulation of bone mineralization
## 45                                                                                           Cdc42 protein signal transduction
## 46                                                                                     negative regulation of bone development
## 47                                                                                           killing by symbiont of host cells
## 48  positive regulation of transcription from RNA polymerase II promoter involved in myocardial precursor cell differentiation
## 49                                                  mesenchymal to epithelial transition involved in metanephros morphogenesis
## 50                                                                                                  fructose metabolic process
## 51                                                                                                              NADH oxidation
## 52                                                                          substrate-dependent cell migration, cell extension
## 53                                                                                                   primary sex determination
## 54                                                     negative regulation of tumor necrosis factor-mediated signaling pathway
## 55                                                                       positive regulation of triglyceride catabolic process
## 56                                                     negative regulation of very-low-density lipoprotein particle remodeling
## 57                                                                                                      neural crest formation
## 58                                                                                        neural crest cell fate specification
## 59                                                                                                           protein oxidation
## 60                                                                                            peptidyl-methionine modification
## 61                                                                                            male germ-line sex determination
## 62                                                                                  regulation of fatty acid metabolic process
## 63                                                                                           triglyceride biosynthetic process
## 64                                                                                  hemolysis by symbiont of host erythrocytes
## 65                                                                                        cerebral cortex tangential migration
## 66                                                                             regulation of intestinal cholesterol absorption
## 67                                                                                              regulation of sterol transport
## 68                                                                             regulation of Cdc42 protein signal transduction
## 69                                                                             negative regulation of interleukin-1 production
## 70                                                                                                          cholesterol efflux
## 71                                                                                            anterior/posterior axon guidance
## 72                                                                                                         phospholipid efflux
## 73                                                                       negative regulation of heterotypic cell-cell adhesion
## 74                                                                                  high-density lipoprotein particle assembly
## 75                                                                      positive regulation of Rho protein signal transduction
## 76                                                                                              sterol transmembrane transport
## 77                                                                                          intrahepatic bile duct development
## 78                                                                                              neural crest cell delamination
## 79                                                                                                     cholesterol homeostasis
## 80                                                                                      regulation of digestive system process
## 81                                                                                                 hemolysis in other organism
## 82                                                                                                             lipid digestion
## 83                                                                      positive regulation of fatty acid biosynthetic process
## 84                                                                                          neutral lipid biosynthetic process
## 85                                                                                      negative regulation of bone remodeling
## 86                                                                         negative regulation of interleukin-1 beta secretion
## 87                                                                                       regulation of lipid catabolic process
## 88                                                                          positive regulation of lipoprotein lipase activity
## 89                                                                                  maintenance of protein location in nucleus
## 90                                                                                       maintenance of mitochondrion location
## 91                                                               cytolysis in other organism involved in symbiotic interaction
## 92                                                     disruption of cells of other organism involved in symbiotic interaction
## 93                                                                                  positive regulation of telomerase activity
## 94                                                                                                    phospholipid homeostasis
## 95                                                                                                    Sertoli cell development
## 96                                                                                            retinal rod cell differentiation
## 97                                                                    negative regulation of cell adhesion molecule production
## 98                                                          epithelial cell proliferation involved in prostatic bud elongation
## 99                                                                                              bronchus cartilage development
## 100                                                                                              trachea cartilage development
## 101                                                                                intestinal epithelial structure maintenance
## 102                                                                       negative regulation of response to cytokine stimulus
## 103                                                            regulation of cell proliferation involved in tissue homeostasis
## 104                                                                     regulation of branching involved in lung morphogenesis
## 105                                                                                             lung smooth muscle development
## 106                                                                                             protein-lipid complex assembly
## 107                                                                                                  thyroid hormone transport
## 108                                                                                                Harderian gland development
## 109                                                                                                         cholesterol import
## 110                                                                                               cellular response to heparin
## 111                                                                                              ureter urothelium development
## 112                                                                                  ureter smooth muscle cell differentiation
## 113                                                                                                       ureter morphogenesis
## 114                                                                                       metanephric nephron tubule formation
## 115                                                                          positive regulation of protein kinase C signaling
## 116                                                    negative regulation of branching involved in ureteric bud morphogenesis
## 117                                                                               regulation of triglyceride metabolic process
## 118                                                                            negative regulation of osteoclast proliferation
## 119                                                               positive regulation of peptidyl-tyrosine autophosphorylation
## 120                                                                            negative regulation of bone trabecula formation
## 121                                                     negative regulation of bone mineralization involved in bone maturation
## 122                                                                              positive regulation of cartilage condensation
## 123                                                                              positive regulation of male gonad development
## 124                                                  positive regulation of cell proliferation involved in heart morphogenesis
## 125                                                               positive regulation of mesenchymal stem cell differentiation
## 126                                                                  negative regulation of mesenchymal cell apoptotic process
## 127                                                                                                    metanephros development
## 128                                                                                               isoprenoid metabolic process
## 129                                                                 modification of morphology or physiology of other organism
## 130                                                                                          cellular protein complex assembly
## 131                                                                                              response to external stimulus
## 132                                                                                                 response to other organism
## 133                                                                                                           tissue migration
## 134                                                                                                              cell adhesion
## 135                                                                                                               angiogenesis
## 136                                                                                                    amine metabolic process
## 137                                                                                defense response to Gram-negative bacterium
## 138                                                                                                 ureteric bud morphogenesis
## 139                                                                                         hepaticobiliary system development
## 140                                                                                               nephron tubule morphogenesis
## 141                                                                                                response to biotic stimulus
## 142                                                                                             phospholipid metabolic process
## 143                                                                                       positive regulation of cell motility
## 144                                                                                                      Wnt signaling pathway
## 145                                                                                   glycerophospholipid biosynthetic process
## 146                                                                                                       kidney morphogenesis
## 147                                                                                                   renal tubule development
## 148                                                                                   monocarboxylic acid biosynthetic process
## 149                                                                                                cellular component assembly
## 150                                                                                       multicellular organismal development
```

```
# GO similarity
library(corrplot)
```

```
## Warning: package 'corrplot' was built under R version 3.2.5
```

```
goListBP <- summary(BP)[,c(1)][1:50] # top 50
goSimMatBP <- mgoSim(goListBP, goListBP, ont="BP", measure="Jiang", organism="chicken", combine=NULL)
corrplot(goSimMatBP, is.corr = FALSE, type="lower", tl.col = "black", tl.cex = 0.8)
```

GO enrichment analysis for **MF**

```
ontology(paraGO) <- "MF"
MF <- hyperGTest(paraGO)
summary(MF)[,c(1,2,7)] # 26
```

```
##        GOMFID      Pvalue
## 1  GO:0030295 0.003340377
## 2  GO:0005504 0.005481988
## 3  GO:0016747 0.007104929
## 4  GO:0030674 0.011162870
## 5  GO:0008237 0.018557479
## 6  GO:0004180 0.023676012
## 7  GO:0008047 0.024166328
## 8  GO:0000254 0.024268824
## 9  GO:0004056 0.024268824
## 10 GO:0004063 0.024268824
## 11 GO:0004366 0.024268824
## 12 GO:0004508 0.024268824
## 13 GO:0004607 0.024268824
## 14 GO:0008035 0.024268824
## 15 GO:0008241 0.024268824
## 16 GO:0019863 0.024268824
## 17 GO:0019864 0.024268824
## 18 GO:0031994 0.024268824
## 19 GO:0031995 0.024268824
## 20 GO:0043184 0.024268824
## 21 GO:0047442 0.024268824
## 22 GO:0060228 0.024268824
## 23 GO:0061609 0.024268824
## 24 GO:0070061 0.024268824
## 25 GO:0070653 0.024268824
## 26 GO:0071814 0.024268824
## 27 GO:0097157 0.024268824
## 28 GO:0016830 0.027496725
## 29 GO:0001758 0.047963417
## 30 GO:0003841 0.047963417
## 31 GO:0004181 0.047963417
## 32 GO:0004332 0.047963417
## 33 GO:0005030 0.047963417
## 34 GO:0015485 0.047963417
## 35 GO:0015643 0.047963417
## 36 GO:0030297 0.047963417
## 37 GO:0031014 0.047963417
## 38 GO:0031210 0.047963417
## 39 GO:0034191 0.047963417
## 40 GO:0048018 0.047963417
## 41 GO:0019207 0.049390009
##                                                                           Term
## 1                                            protein kinase activator activity
## 2                                                           fatty acid binding
## 3  transferase activity, transferring acyl groups other than amino-acyl groups
## 4                                                    protein binding, bridging
## 5                                                    metallopeptidase activity
## 6                                                    carboxypeptidase activity
## 7                                                    enzyme activator activity
## 8                                            C-4 methylsterol oxidase activity
## 9                                             argininosuccinate lyase activity
## 10                                             aryldialkylphosphatase activity
## 11                             glycerol-3-phosphate O-acyltransferase activity
## 12                                     steroid 17-alpha-monooxygenase activity
## 13                       phosphatidylcholine-sterol O-acyltransferase activity
## 14                                   high-density lipoprotein particle binding
## 15                                               peptidyl-dipeptidase activity
## 16                                                                 IgE binding
## 17                                                                 IgG binding
## 18                                        insulin-like growth factor I binding
## 19                                       insulin-like growth factor II binding
## 20                       vascular endothelial growth factor receptor 2 binding
## 21                              17-alpha-hydroxyprogesterone aldolase activity
## 22             phosphatidylcholine-sterol O-acyltransferase activator activity
## 23                                      fructose-1-phosphate aldolase activity
## 24                                                            fructose binding
## 25                          high-density lipoprotein particle receptor binding
## 26                                               protein-lipid complex binding
## 27                                                   pre-mRNA intronic binding
## 28                                                carbon-carbon lyase activity
## 29                                              retinal dehydrogenase activity
## 30                       1-acylglycerol-3-phosphate O-acyltransferase activity
## 31                                            metallocarboxypeptidase activity
## 32                                     fructose-bisphosphate aldolase activity
## 33                                              neurotrophin receptor activity
## 34                                                         cholesterol binding
## 35                                                     toxic substance binding
## 36           transmembrane receptor protein tyrosine kinase activator activity
## 37                                                          troponin T binding
## 38                                                 phosphatidylcholine binding
## 39                                         apolipoprotein A-I receptor binding
## 40                                                   receptor agonist activity
## 41                                                   kinase regulator activity
```

```
# GO similarity
goListMF <- summary(MF)[,c(1)]
goSimMatMF <- mgoSim(goListMF, goListMF, ont="MF", measure="Jiang", organism="chicken", combine=NULL)
corrplot(goSimMatMF, is.corr = FALSE, type="lower", tl.col = "black", tl.cex = 0.8)
```

GO enrichment analysis for **CC**

```
ontology(paraGO) <- "CC"
CC <- hyperGTest(paraGO)
summary(CC)[,c(1,2,7)] # 7
```

```
##       GOCCID       Pvalue                                        Term
## 1 GO:0005576 1.420690e-08                        extracellular region
## 2 GO:0005577 8.363040e-04                          fibrinogen complex
## 3 GO:0060417 2.461834e-03                                        yolk
## 4 GO:0005604 6.813459e-03                           basement membrane
## 5 GO:0005578 1.656586e-02          proteinaceous extracellular matrix
## 6 GO:0034366 2.917772e-02 spherical high-density lipoprotein particle
## 7 GO:0030017 3.083484e-02                                   sarcomere
```

```
# GO similarity
goListCC <- summary(CC)[,c(1)]
goSimMatCC <- mgoSim(goListCC, goListCC, ont="CC", measure="Jiang", organism="chicken", combine=NULL)
corrplot(goSimMatCC[-3,-3], is.corr = FALSE, type="lower", tl.col = "black", tl.cex = 0.8)
```

## 4. MeSH enrichment analysis

Then, we perform a MeSH ORA for the category **Chemicals and Drugs** by setting ‘category=“D”’.

```
library(meshr)
library(MeSH.db)
library("MeSH.Gga.eg.db")
meshParams <- new("MeSHHyperGParams", geneIds = my.geneID3[,2], universeGeneIds = univ.geneID3[,2], 
                  annotation = "MeSH.Gga.eg.db", category = "D", database = "gene2pubmed", 
                  pvalueCutoff = 0.05, pAdjust = "none")
meshR <- meshHyperGTest(meshParams)
summary(meshR)[!duplicated(summary(meshR)[,7]),c(1,2,7)] # 179
```

```
##       MESHID       Pvalue
## 3460 D006023 7.968146e-09
## 4557 D009414 1.000085e-05
## 4120 D008565 6.593672e-05
## 3875 D008074 1.055079e-04
## 6864 D012697 2.578723e-04
## 9410 D051153 3.350551e-04
## 5148 D009439 3.434309e-04
## 9880 D060528 3.434309e-04
## 7250 D014874 3.523914e-04
## 7777 D017931 4.006199e-04
## 9258 D040121 5.079934e-04
## 3929 D008562 5.545764e-04
## 9239 D039961 9.703630e-04
## 405  D000806 1.044018e-03
## 2196 D004549 1.044018e-03
## 5226 D011228 1.044018e-03
## 7772 D017038 1.044018e-03
## 9720 D054387 1.044018e-03
## 9748 D054839 1.044018e-03
## 7351 D015850 1.133017e-03
## 474  D002352 1.243176e-03
## 306  D000596 1.725201e-03
## 7634 D016326 1.860294e-03
## 993  D003851 2.062091e-03
## 6914 D012709 2.062091e-03
## 8393 D019718 2.062091e-03
## 9303 D045702 2.062091e-03
## 5275 D011506 2.132027e-03
## 898  D003459 3.258086e-03
## 1    D000117 3.394179e-03
## 408  D001053 3.394179e-03
## 2414 D005419 3.394179e-03
## 5142 D009438 3.394179e-03
## 6928 D013381 3.394179e-03
## 9214 D039942 3.394179e-03
## 9297 D045683 3.394179e-03
## 9340 D050556 3.678819e-03
## 8425 D020033 4.006211e-03
## 5702 D011518 4.566675e-03
## 1009 D004247 4.893274e-03
## 2395 D005340 5.028200e-03
## 3642 D007073 5.028200e-03
## 5220 D011108 5.028200e-03
## 6838 D012177 5.028200e-03
## 6942 D013917 5.028200e-03
## 8406 D019743 5.028200e-03
## 9231 D039943 5.028200e-03
## 9395 D050796 5.028200e-03
## 9702 D054377 5.028200e-03
## 6185 D011993 5.480342e-03
## 4639 D009419 5.618871e-03
## 8327 D019063 5.916373e-03
## 434  D001054 6.952412e-03
## 445  D001141 6.952412e-03
## 459  D001216 6.952412e-03
## 857  D002630 6.952412e-03
## 9327 D047493 6.952412e-03
## 9896 D064235 6.952412e-03
## 9035 D036341 7.745054e-03
## 6456 D011994 7.816209e-03
## 7048 D014357 7.962759e-03
## 3897 D008079 9.155405e-03
## 9287 D043484 9.155405e-03
## 9762 D055748 1.114071e-02
## 999  D004054 1.162609e-02
## 6933 D013739 1.162609e-02
## 7228 D014801 1.162609e-02
## 9726 D054834 1.162609e-02
## 5955 D011956 1.238517e-02
## 3649 D007136 1.416071e-02
## 8932 D029867 1.416071e-02
## 7144 D014443 1.430798e-02
## 8696 D020662 1.435371e-02
## 8708 D020746 1.435371e-02
## 8372 D019151 1.732779e-02
## 8783 D024043 1.732779e-02
## 9641 D051780 1.732779e-02
## 404  D000726 1.880721e-02
## 472  D002122 1.880721e-02
## 473  D002216 1.880721e-02
## 864  D002785 1.880721e-02
## 868  D002839 1.880721e-02
## 893  D003165 1.880721e-02
## 894  D003174 1.880721e-02
## 895  D003176 1.880721e-02
## 956  D003573 1.880721e-02
## 992  D003620 1.880721e-02
## 2202 D004851 1.880721e-02
## 2203 D004852 1.880721e-02
## 2394 D005338 1.880721e-02
## 2412 D005344 1.880721e-02
## 2413 D005345 1.880721e-02
## 2419 D005485 1.880721e-02
## 3872 D007862 1.880721e-02
## 5157 D009543 1.880721e-02
## 5158 D009961 1.880721e-02
## 5217 D010127 1.880721e-02
## 5218 D010416 1.880721e-02
## 5219 D010928 1.880721e-02
## 6863 D012450 1.880721e-02
## 6926 D013196 1.880721e-02
## 6927 D013254 1.880721e-02
## 6952 D014049 1.880721e-02
## 7140 D014359 1.880721e-02
## 7254 D015728 1.880721e-02
## 7389 D016173 1.880721e-02
## 7776 D017367 1.880721e-02
## 8391 D019307 1.880721e-02
## 8392 D019426 1.880721e-02
## 8720 D020781 1.880721e-02
## 8721 D020913 1.880721e-02
## 9034 D036082 1.880721e-02
## 9286 D043303 1.880721e-02
## 9339 D050494 1.880721e-02
## 9407 D050890 1.880721e-02
## 9639 D051199 1.880721e-02
## 9640 D051398 1.880721e-02
## 9672 D053606 1.880721e-02
## 9699 D053676 1.880721e-02
## 9700 D053779 1.880721e-02
## 9724 D054589 1.880721e-02
## 9879 D059002 1.880721e-02
## 261  D000242 2.005160e-02
## 6166 D011991 2.053816e-02
## 9193 D038681 2.053816e-02
## 9673 D053674 2.053816e-02
## 2204 D005136 2.228595e-02
## 5159 D010047 2.397497e-02
## 8    D000199 2.489505e-02
## 3747 D007527 2.509153e-02
## 8309 D018925 2.762862e-02
## 8754 D024002 2.762862e-02
## 7259 D015816 2.764424e-02
## 7394 D016212 2.967478e-02
## 5250 D011374 3.148981e-02
## 6953 D014212 3.225025e-02
## 9814 D055758 3.420595e-02
## 300  D000516 3.726203e-02
## 467  D001789 3.726203e-02
## 469  D002070 3.726203e-02
## 865  D002794 3.726203e-02
## 950  D003486 3.726203e-02
## 989  D003580 3.726203e-02
## 2420 D005634 3.726203e-02
## 3867 D007703 3.726203e-02
## 3873 D008043 3.726203e-02
## 4555 D009251 3.726203e-02
## 6859 D012337 3.726203e-02
## 7141 D014361 3.726203e-02
## 7226 D014760 3.726203e-02
## 7252 D015230 3.726203e-02
## 7255 D015786 3.726203e-02
## 7387 D015926 3.726203e-02
## 7391 D016186 3.726203e-02
## 8386 D019272 3.726203e-02
## 8389 D019301 3.726203e-02
## 8419 D019809 3.726203e-02
## 8780 D024003 3.726203e-02
## 8797 D024082 3.726203e-02
## 8832 D028884 3.726203e-02
## 9211 D038702 3.726203e-02
## 9295 D044139 3.726203e-02
## 9336 D050486 3.726203e-02
## 9390 D050784 3.726203e-02
## 9403 D050822 3.726203e-02
## 9405 D050882 3.726203e-02
## 9670 D052244 3.726203e-02
## 9811 D055751 3.726203e-02
## 9911 D064236 3.726203e-02
## 869  D002918 3.979893e-02
## 8722 D023181 3.979893e-02
## 9307 D047428 3.979893e-02
## 2424 D005819 4.084448e-02
## 8251 D018160 4.409130e-02
## 2081 D004262 4.461181e-02
## 8834 D029721 4.656610e-02
## 958  D003577 4.883317e-02
## 8799 D026901 4.883317e-02
## 9605 D051155 4.883317e-02
##                                                     MESHTERM
## 3460                                           Glycoproteins
## 4557                                    Nerve Growth Factors
## 4120                                       Membrane Proteins
## 3875                                            Lipoproteins
## 6864                                   Serine Endopeptidases
## 9410                                            Wnt Proteins
## 5148                                           Neuraminidase
## 9880                                            Wnt4 Protein
## 7250                              Water Pollutants, Chemical
## 7777                                             DNA Primers
## 9258                                           Semaphorin-3A
## 3929                                  Membrane Glycoproteins
## 9239                                             Semaphorins
## 405                 Angiotensin-Converting Enzyme Inhibitors
## 2196                                                 Elastin
## 5226                                              Prealbumin
## 7772 Mannosyl-Glycoprotein Endo-beta-N-Acetylglucosaminidase
## 9720                                         Receptors, CXCR
## 9748                        Retinol-Binding Proteins, Plasma
## 7351                                           Interleukin-6
## 474                                         Carrier Proteins
## 306                                              Amino Acids
## 7634                           Extracellular Matrix Proteins
## 993                                       Deoxyribonucleases
## 6914                                           Serum Albumin
## 8393                                        Receptors, CXCR4
## 9303                                 Proprotein Convertase 5
## 5275                                                Proteins
## 898                                              Crystallins
## 1                                          Acetylglucosamine
## 408                                          Apolipoproteins
## 2414                                              Flavonoids
## 5142                                        Neuraminic Acids
## 6928                                             Subtilisins
## 9214                                            Neuropilin-1
## 9297                                                   Furin
## 9340                             Fatty Acid-Binding Proteins
## 8425                                        Protein Isoforms
## 5702                                 Proto-Oncogene Proteins
## 1009                                                     DNA
## 2395                                              Fibrinogen
## 3642                                        Immunoglobulin E
## 5220                                                Polymers
## 6838                                Retinol-Binding Proteins
## 6942                                                Thrombin
## 8406                                         Chemokines, CXC
## 9231                                            Neuropilin-2
## 9395                              STAT3 Transcription Factor
## 9702                                        Chemokine CXCL12
## 6185                             Recombinant Fusion Proteins
## 4639                                   Nerve Tissue Proteins
## 8327                                                Tenascin
## 434                                        Apolipoproteins A
## 445                                                Aromatase
## 459                                               Asparagine
## 857                                      Chemotactic Factors
## 9327                                              PPAR alpha
## 9896                                       Matrilin Proteins
## 9035           Intercellular Signaling Peptides and Proteins
## 6456                                    Recombinant Proteins
## 7048                                                 Trypsin
## 3897                                      Lipoproteins, VLDL
## 9287                                  Proprotein Convertases
## 9762                             SOXB1 Transcription Factors
## 999                                       Diethylstilbestrol
## 6933                                            Testosterone
## 7228                                               Vitamin A
## 9726                                              Lipocalins
## 5955                                 Receptors, Cell Surface
## 3649                                         Immunoglobulins
## 8932                                        Xenopus Proteins
## 7144                                                Tyrosine
## 8696                     Guanine Nucleotide Exchange Factors
## 8708                                Calcium Channels, L-Type
## 8372                                         Peptide Library
## 8783                                        Collagen Type II
## 9641             Sterol Regulatory Element Binding Protein 1
## 404                                     Androgen Antagonists
## 472                                         Calcium Chloride
## 473                                                Captopril
## 864                                 Sterol O-Acyltransferase
## 868                                                 Chromans
## 893                               Complement System Proteins
## 894                       Complement C1 Inactivator Proteins
## 895                                            Complement C3
## 956                                       Cytochrome b Group
## 992                                               Dantrolene
## 2202                                      Epoxide Hydrolases
## 2203                                         Epoxy Compounds
## 2394                  Fibrin Fibrinogen Degradation Products
## 2412                                        Fibrinopeptide A
## 2413                                        Fibrinopeptide B
## 2419                                               Flutamide
## 3872            Phosphatidylcholine-Sterol O-Acyltransferase
## 5157                                              Nifedipine
## 5158                                             Orosomucoid
## 5217                                    P Blood-Group System
## 5218                                       Pentachlorophenol
## 5219                                      Placental Lactogen
## 6863                                           Safflower Oil
## 6926                                     Dihydrotestosterone
## 6927                            Steroid 17-alpha-Hydroxylase
## 6952                                             Tolperisone
## 7140                     Trypsin Inhibitor, Kazal Pancreatic
## 7254                                          Receptors, HIV
## 7389                    Macrophage Colony-Stimulating Factor
## 7776                             Serotonin Uptake Inhibitors
## 8391           1-(5-Isoquinolinesulfonyl)-2-Methylpiperazine
## 8392                                              Integrases
## 8720                              Matrix Metalloproteinase 1
## 8721                                             Perindopril
## 9034                                         Receptor, EphA1
## 9286                                  Aryldialkylphosphatase
## 9339              Vesicular Acetylcholine Transport Proteins
## 9407                               HSP30 Heat-Shock Proteins
## 9639                                    Toll-Like Receptor 7
## 9640                                             Aquaporin 1
## 9672                                      Receptors, OSM-LIF
## 9699                                               Syntenins
## 9700                        Latent TGF-beta Binding Proteins
## 9724       Protein Tyrosine Phosphatase, Non-Receptor Type 3
## 9879                           Androgen Receptor Antagonists
## 261                                               Cyclic AMP
## 6166                                        Receptors, Virus
## 9193                                             Follistatin
## 9673                                               Aggrecans
## 2204                                            Eye Proteins
## 5159                                               Ovalbumin
## 8                                                     Actins
## 3747                                              Isoenzymes
## 8309                                              Chemokines
## 8754                                           HMGB Proteins
## 7259                       Cell Adhesion Molecules, Neuronal
## 7394                         Transforming Growth Factor beta
## 5250                                            Progesterone
## 6953                                               Tretinoin
## 9814                               SOX9 Transcription Factor
## 300                                           alpha-Amylases
## 467                                     Blood Group Antigens
## 469                                               Butadienes
## 865                                                  Choline
## 950                                                 Cyanides
## 989                                              Cytochromes
## 2420                          Fructose-Bisphosphate Aldolase
## 3867                                  Peptidyl-Dipeptidase A
## 3873                                             Linseed Oil
## 4555                        NADPH-Ferrihemoprotein Reductase
## 6859                                     RNA, Ribosomal, 18S
## 7141                                      Trypsin Inhibitors
## 7226                                   Viral Fusion Proteins
## 7252                                        Prostaglandin D2
## 7255                                          Cytochromes b5
## 7387                                          Complement C3a
## 7391          Receptor, Macrophage Colony-Stimulating Factor
## 8386                                      Leukocyte Elastase
## 8389                                              Oleic Acid
## 8419                                            Streptavidin
## 8780                                           HMGA Proteins
## 8797                                       Collagen Type XII
## 8832                                            Karyopherins
## 9211                            Follistatin-Related Proteins
## 9295                           Receptor, Angiotensin, Type 2
## 9336            Serotonin Plasma Membrane Transport Proteins
## 9390                          Proto-Oncogene Protein c-ets-1
## 9403                                 Cytokine Receptor gp130
## 9405                                    CREB-Binding Protein
## 9670                                    Endocrine Disruptors
## 9811                              SOXC Transcription Factors
## 9911                     Cartilage Oligomeric Matrix Protein
## 869                                             Chymotrypsin
## 8722                         Antimicrobial Cationic Peptides
## 9307                               Protein Kinase Inhibitors
## 2424                                         Genetic Markers
## 8251                      Receptors, Cytoplasmic and Nuclear
## 2081                                 DNA Restriction Enzymes
## 8834                                     Drosophila Proteins
## 958                           Cytochrome P-450 Enzyme System
## 8799                             Membrane Transport Proteins
## 9605                                            Wnt1 Protein
```

```
# MeSH similarity
library("MeSHSim")
headingListD <- summary(meshR)[!duplicated(summary(meshR)[,7]),c(7)]
meshSimMatD <- mheadingSim(headingListD, headingListD, method="JC")
rownames(meshSimMatD) <- colnames(meshSimMatD) <- summary(meshR)[!duplicated(summary(meshR)[,7]),c(1)]
indexD <- which(meshSimMatD > 0.35 & meshSimMatD != 1, arr.ind = TRUE)
corrplot(meshSimMatD[unique(rownames(meshSimMatD)[indexD[,1]]), 
                     unique(rownames(meshSimMatD)[indexD[,1]])], is.corr = FALSE, type="lower", 
         tl.col = "black", tl.cex = 0.8)
```

Switching to a different category is easily done by the ‘category<-’ function. Here, we use **Diseases** (category = “C”).

```
category(meshParams) <- "C"
meshR <- meshHyperGTest(meshParams)
summary(meshR)[!duplicated(summary(meshR)[,7]),c(1,2,7)] # 29
```

```
##      MESHID       Pvalue                            MESHTERM
## 113 D009521 0.0005079934                   Newcastle Disease
## 7   D003333 0.0010440180            Coronaviridae Infections
## 123 D012088 0.0020620909               Reoviridae Infections
## 108 D009336 0.0033941788                            Necrosis
## 130 D014802 0.0033941788                Vitamin A Deficiency
## 12  D004195 0.0042746874              Disease Models, Animal
## 3   D000419 0.0188072082                         Albuminuria
## 4   D001249 0.0188072082                              Asthma
## 5   D002114 0.0188072082                          Calcinosis
## 6   D003233 0.0188072082            Conjunctivitis, Allergic
## 83  D005923 0.0188072082 Glomerulosclerosis, Focal Segmental
## 86  D006349 0.0188072082                Heart Valve Diseases
## 89  D006967 0.0188072082                    Hypersensitivity
## 92  D007057 0.0188072082                          Ichthyosis
## 127 D012221 0.0188072082       Rhinitis, Allergic, Perennial
## 136 D014855 0.0188072082              Wallerian Degeneration
## 144 D058186 0.0188072082                 Acute Kidney Injury
## 93  D007249 0.0205381626                        Inflammation
## 1   D000130 0.0372620251                      Achondroplasia
## 10  D003924 0.0372620251           Diabetes Mellitus, Type 2
## 81  D004535 0.0372620251              Ehlers-Danlos Syndrome
## 84  D006345 0.0372620251   Heart Septal Defects, Ventricular
## 87  D006965 0.0372620251                         Hyperplasia
## 90  D007039 0.0372620251                       Hypotrichosis
## 106 D007842 0.0372620251                           Lathyrism
## 128 D013733 0.0372620251                 Testicular Diseases
## 137 D015427 0.0372620251                  Reperfusion Injury
## 140 D020347 0.0372620251                           Lithiasis
## 142 D021181 0.0372620251                Egg Hypersensitivity
```

```
# MeSH similarity
headingListC <- summary(meshR)[!duplicated(summary(meshR)[,7]),c(7)]
meshSimMatC <- mheadingSim(headingListC, headingListC, method="JC")
rownames(meshSimMatC) <- colnames(meshSimMatC) <- summary(meshR)[!duplicated(summary(meshR)[,7]),c(1)]
indexC <- which(meshSimMatC > 0.025 & meshSimMatC != 1, arr.ind = TRUE)
corrplot(meshSimMatC, is.corr = FALSE, type="lower", tl.col = "black", tl.cex = 0.8)
```

MeSH ORA for **Anatomy** (category = “A”).

```
category(meshParams) <- "A"
meshR <- meshHyperGTest(meshParams)
summary(meshR)[!duplicated(summary(meshR)[,7]),c(1,2,7)] # 66
```

```
##        MESHID       Pvalue                        MESHTERM
## 7619  D008099 3.523638e-07                           Liver
## 10532 D018699 2.594517e-05                 Coated Vesicles
## 6825  D005121 1.330416e-04                     Extremities
## 9173  D009900 1.417543e-04                     Optic Nerve
## 417   D001853 1.858506e-04                     Bone Marrow
## 8062  D008334 2.176694e-04                        Mandible
## 8070  D008648 2.679905e-04                        Mesoderm
## 10536 D018878 2.881693e-04                       Limb Buds
## 1476  D002642 3.705599e-04                    Chick Embryo
## 453   D002478 4.049824e-04                 Cells, Cultured
## 9722  D013116 4.361241e-04                     Spinal Cord
## 7448  D007668 6.293158e-04                          Kidney
## 6599  D004530 1.275901e-03                        Egg Yolk
## 7069  D005123 2.154362e-03                             Eye
## 8487  D009432 3.029153e-03                    Neural Crest
## 6377  D002889 3.394179e-03      Chromosomes, Human, Pair 2
## 6472  D004475 3.465261e-03                        Ectoderm
## 9104  D009865 3.660892e-03                         Oocytes
## 8386  D008862 5.028200e-03               Microsomes, Liver
## 11459 D059630 5.916373e-03       Mesenchymal Stromal Cells
## 7180  D005855 6.952412e-03                     Germ Layers
## 11025 D020439 7.812322e-03                    Growth Cones
## 8688  D009474 8.991802e-03                         Neurons
## 6726  D005109 9.570570e-03            Extracellular Matrix
## 11368 D053595 1.114071e-02            Embryonic Stem Cells
## 6717  D004727 1.162609e-02                     Endothelium
## 7187  D006107 1.242174e-02                 Granulosa Cells
## 9207  D010053 1.369892e-02                           Ovary
## 9338  D012160 1.398331e-02                          Retina
## 9901  D013154 1.491848e-02                          Spleen
## 11077 D032446 1.522718e-02                       Myoblasts
## 11115 D050153 1.522718e-02                   Abdominal Fat
## 10130 D013564 1.732779e-02      Sympathetic Nervous System
## 10804 D019556 1.774163e-02                       COS Cells
## 25    D001133 1.880721e-02        Bones of Upper Extremity
## 26    D001339 1.880721e-02 Autonomic Fibers, Preganglionic
## 6383  D003228 1.880721e-02                     Conjunctiva
## 6384  D003239 1.880721e-02         Connective Tissue Cells
## 8393  D008943 1.880721e-02                    Mitral Valve
## 9335  D011708 1.880721e-02                         Pylorus
## 10483 D014261 1.880721e-02                 Tricuspid Valve
## 11367 D050527 1.880721e-02                 Mesangial Cells
## 11400 D054258 2.053816e-02                    Neural Plate
## 10045 D013477 2.347781e-02              Superior Colliculi
## 7576  D007962 2.368206e-02                      Leukocytes
## 27    D001808 2.397497e-02                   Blood Vessels
## 9191  D010006 2.397497e-02                     Osteoblasts
## 10467 D013799 2.397497e-02                     Theca Cells
## 10145 D013710 2.764424e-02                         Tendons
## 6385  D003599 2.914083e-02                    Cytoskeleton
## 7158  D005145 3.148981e-02                            Face
## 10671 D019170 3.215174e-02                         Somites
## 10188 D013737 3.235859e-02                          Testis
## 11412 D054259 3.655662e-02                     Neural Tube
## 1389  D002479 3.726203e-02                Inclusion Bodies
## 9333  D010949 3.726203e-02                          Plasma
## 9718  D012540 3.726203e-02                         Scapula
## 9720  D012708 3.726203e-02                   Sertoli Cells
## 10481 D014066 3.726203e-02                 Palatine Tonsil
## 10484 D016548 3.898816e-02                  Prosencephalon
## 8395  D009046 4.084448e-02                   Motor Neurons
## 1393  D002490 4.270459e-02          Central Nervous System
## 8004  D008264 4.409130e-02                     Macrophages
## 1     D001011 4.422957e-02                           Aorta
## 7221  D006321 4.617024e-02                           Heart
## 43    D001842 4.950968e-02                  Bone and Bones
```

```
headingListA <- summary(meshR)[!duplicated(summary(meshR)[,7]),c(7)]
meshSimMatA <- mheadingSim(headingListA, headingListA, method="JC")
rownames(meshSimMatA) <- colnames(meshSimMatA) <- summary(meshR)[!duplicated(summary(meshR)[,7]),c(1)]
indexA <- which(meshSimMatA > 0.15 & meshSimMatA != 1, arr.ind = TRUE)
corrplot(meshSimMatA[unique(rownames(meshSimMatA)[indexA[,1]]),
                     unique(rownames(meshSimMatA)[indexA[,1]])], is.corr = FALSE, type="lower", 
         tl.col = "black", tl.cex = 0.8)
```

MeSH ORA for **Phenomena and Processes** (category = “G”).

```
category(meshParams) <- "G"
meshR <- meshHyperGTest(meshParams)
summary(meshR)[!duplicated(summary(meshR)[,7]),c(1,2,7)] # 61
```

```
##        MESHID       Pvalue                                  MESHTERM
## 277   D000595 3.389919e-09                       Amino Acid Sequence
## 18384 D015870 4.028123e-07                           Gene Expression
## 19403 D017386 5.978909e-06             Sequence Homology, Amino Acid
## 15835 D013997 3.031943e-04                              Time Factors
## 13592 D011485 4.970937e-04                           Protein Binding
## 4369  D001483 1.883463e-03                             Base Sequence
## 15724 D013047 2.053056e-03          Specific Pathogen-Free Organisms
## 13351 D007814 2.062091e-03                                     Larva
## 9053  D001923 3.378975e-03                           Brain Chemistry
## 9915  D002889 3.394179e-03                Chromosomes, Human, Pair 2
## 13365 D009336 3.394179e-03                                  Necrosis
## 13370 D009693 4.371130e-03                Nucleic Acid Hybridization
## 16324 D015398 5.256284e-03                       Signal Transduction
## 14505 D011487 5.278250e-03                      Protein Conformation
## 15091 D013045 6.709171e-03                       Species Specificity
## 11504 D005838 7.745054e-03                                  Genotype
## 14816 D011489 7.774415e-03                      Protein Denaturation
## 1     D000375 8.476071e-03                                     Aging
## 16287 D014961 8.818046e-03                         X-Ray Diffraction
## 4359  D001343 9.155405e-03                                 Autophagy
## 9958  D005075 9.747289e-03                      Biological Evolution
## 10115 D005091 1.119189e-02                                     Exons
## 8997  D001667 1.157491e-02                      Binding, Competitive
## 13355 D008390 1.162609e-02                             Markov Chains
## 23388 D054338 1.162609e-02                 Cell Transdifferentiation
## 18217 D015854 1.191336e-02                             Up-Regulation
## 20925 D018507 1.289404e-02 Gene Expression Regulation, Developmental
## 13295 D006031 1.335579e-02                             Glycosylation
## 20762 D017433 1.430758e-02              Protein Structure, Secondary
## 23201 D019943 1.430798e-02                   Amino Acid Substitution
## 23374 D040681 1.435371e-02              Structural Homology, Protein
## 17605 D015723 1.445543e-02                              Gene Library
## 23307 D024510 1.633875e-02                        Muscle Development
## 8362  D001665 1.720409e-02                             Binding Sites
## 13280 D005865 1.732779e-02                           Gestational Age
## 22928 D019151 1.732779e-02                           Peptide Library
## 276   D000478 1.880721e-02                                Alkylation
## 9957  D004306 1.880721e-02   Dose-Response Relationship, Immunologic
## 14855 D012079 1.880721e-02                         Renal Circulation
## 14856 D012119 1.880721e-02                               Respiration
## 19175 D016163 1.880721e-02                                Genes, DCC
## 19176 D017102 1.880721e-02                          Fracture Healing
## 23306 D024282 1.880721e-02                                Genes, sry
## 15742 D013312 2.053816e-02                     Stress, Physiological
## 22942 D019521 2.187740e-02                           Body Patterning
## 9921  D004032 2.368206e-02                                      Diet
## 23399 D055503 2.368206e-02                   Protein Multimerization
## 19179 D017209 2.729612e-02                                 Apoptosis
## 14857 D012727 2.762862e-02                       Sex Characteristics
## 15006 D012741 2.762862e-02                         Sexual Maturation
## 9122  D002454 2.953272e-02                      Cell Differentiation
## 15779 D013696 2.974933e-02                               Temperature
## 22850 D018628 3.148981e-02                               Gene Dosage
## 14982 D012733 3.554951e-02                       Sex Differentiation
## 23261 D020013 3.655662e-02                         Calcium Signaling
## 22847 D018598 3.726203e-02                     Minisatellite Repeats
## 23386 D054337 3.726203e-02                    Cell Dedifferentiation
## 10468 D005819 4.084448e-02                           Genetic Markers
## 13572 D010012 4.883317e-02                              Osteogenesis
## 22872 D018919 4.950968e-02           Neovascularization, Physiologic
```

```
headingListG <- summary(meshR)[!duplicated(summary(meshR)[,7]),c(7)]
meshSimMatG <- mheadingSim(headingListG, headingListG, method="JC")
rownames(meshSimMatG) <- colnames(meshSimMatG) <- summary(meshR)[!duplicated(summary(meshR)[,7]),c(1)]
indexG <- which(meshSimMatG > 0.1 & meshSimMatG != 1, arr.ind = TRUE)
corrplot(meshSimMatG[unique(rownames(meshSimMatG)[indexG[,1]]), 
                     unique(rownames(meshSimMatG)[indexG[,1]])], is.corr = FALSE, type="lower", 
         tl.col = "black", tl.cex = 0.8)
```

## 5. Session Information

```
sessionInfo()
```

```
## R version 3.2.4 (2016-03-10)
## Platform: x86_64-apple-darwin13.4.0 (64-bit)
## Running under: OS X 10.10.5 (Yosemite)
## 
## locale:
## [1] en_US.UTF-8/en_US.UTF-8/en_US.UTF-8/C/en_US.UTF-8/en_US.UTF-8
## 
## attached base packages:
##  [1] grid      parallel  stats4    stats     graphics  grDevices utils    
##  [8] datasets  methods   base     
## 
## other attached packages:
##  [1] MeSHSim_1.2.0            MeSH.Gga.eg.db_1.5.0    
##  [3] meshr_1.6.2              MeSH.Syn.eg.db_1.5.0    
##  [5] MeSH.Bsu.168.eg.db_1.5.0 MeSH.Aca.eg.db_1.5.0    
##  [7] MeSH.Hsa.eg.db_1.5.0     MeSH.PCR.db_1.5.0       
##  [9] MeSH.AOR.db_1.5.0        MeSH.db_1.5.0           
## [11] MeSHDbi_1.6.0            org.Hs.eg.db_3.2.3      
## [13] cummeRbund_2.12.1        Gviz_1.14.7             
## [15] rtracklayer_1.30.4       GenomicRanges_1.22.4    
## [17] GenomeInfoDb_1.6.3       fastcluster_1.1.20      
## [19] reshape2_1.4.1           ggplot2_2.1.0           
## [21] fdrtool_1.2.15           corrplot_0.77           
## [23] GOSemSim_1.28.2          GOstats_2.36.0          
## [25] graph_1.48.0             Category_2.36.0         
## [27] GO.db_3.2.2              Matrix_1.2-5            
## [29] org.Gg.eg.db_3.2.3       RSQLite_1.0.0           
## [31] DBI_0.3.1                AnnotationDbi_1.32.3    
## [33] IRanges_2.4.8            S4Vectors_0.8.11        
## [35] Biobase_2.30.0           BiocGenerics_0.16.1     
## [37] biomaRt_2.26.1          
## 
## loaded via a namespace (and not attached):
##  [1] bitops_1.0-6               matrixStats_0.50.2        
##  [3] RColorBrewer_1.1-2         tools_3.2.4               
##  [5] rpart_4.1-10               Hmisc_3.17-3              
##  [7] colorspace_1.2-6           nnet_7.3-12               
##  [9] gridExtra_2.2.1            formatR_1.3               
## [11] scales_0.4.0               genefilter_1.52.1         
## [13] RBGL_1.46.0                stringr_1.0.0             
## [15] digest_0.6.9               Rsamtools_1.22.0          
## [17] foreign_0.8-66             rmarkdown_0.9.5           
## [19] AnnotationForge_1.12.2     XVector_0.10.0            
## [21] dichromat_2.0-0            htmltools_0.3.5           
## [23] BSgenome_1.38.0            BiocParallel_1.4.3        
## [25] acepack_1.3-3.3            VariantAnnotation_1.16.4  
## [27] RCurl_1.95-4.8             magrittr_1.5              
## [29] Formula_1.2-1              futile.logger_1.4.1       
## [31] Rcpp_0.12.4                munsell_0.4.3             
## [33] stringi_1.0-1              yaml_2.1.13               
## [35] SummarizedExperiment_1.0.2 zlibbioc_1.16.0           
## [37] plyr_1.8.3                 lattice_0.20-33           
## [39] Biostrings_2.38.4          splines_3.2.4             
## [41] GenomicFeatures_1.22.13    annotate_1.48.0           
## [43] knitr_1.12.3               futile.options_1.0.0      
## [45] XML_3.98-1.4               evaluate_0.8.3            
## [47] biovizBase_1.18.0          latticeExtra_0.6-28       
## [49] lambda.r_1.1.7             gtable_0.2.0              
## [51] xtable_1.8-2               survival_2.39-2           
## [53] GenomicAlignments_1.6.3    cluster_2.0.4             
## [55] GSEABase_1.32.0
```
