# Supplementary material for: MeSH-Informed Enrichment Analysis and MeSH-Guided Semantic Similarity Among Functional Terms and Gene Products in Chicken
Source: G3 (Bethesda). 2016 Jun 2;6(8):2447–53. doi: 10.1534/g3.116.031096 (PMC4978898; doi:10.1534/g3.116.031096)
Supplement: Supplemental Material [file supp_g3.116.031096_FileS2.zip › FileS2.html]

MeSH over-representation analysis (Selective sweep data)


# MeSH over-representation analysis (Selective sweep data)

## 1. Create a vector of background genes

We first create a vector of background genes.

```
library(biomaRt)
## access to biomaRt
#mart <- useMart(biomart = "ensembl", dataset = "ggallus_gene_ensembl")
mart <- useMart(biomart = "ENSEMBL_MART_ENSEMBL", dataset = "ggallus_gene_ensembl", 
                host = "www.ensembl.org")
univ.geneID <- getBM(attributes=c("ensembl_gene_id", "entrezgene", 
                                  "hgnc_symbol"), mart = mart) # 17680 
## remove genes with no corresponding Entrez Gene ID
univ.geneID2 <- univ.geneID[!is.na(univ.geneID[,2]),] # 14124 
## remove duplicated Entrez Gene ID
univ.geneID3 <- univ.geneID2[ !duplicated(univ.geneID2[,2]),] # 13984
```

# 2. Create a vector of selected genes

Secondly, we create a vector of significant genes by reading the input file.

```
## read data
my.geneID <- read.table("allSelectedGenes.txt", colClasses = c("NULL", "character", "NULL"), 
                               sep="\t", header=TRUE) # 397
colnames(my.geneID) <- "ensembl_gene_id"
## merge two files
my.geneID2 <- merge(my.geneID, univ.geneID3, by ="ensembl_gene_id") # 352
## remove duplicated Entrez Gene ID
my.geneID3 <- my.geneID2[ !duplicated(my.geneID2$entrezgene),] # 352
```

## 3. GO enrichment analysis

We perform a GO analysis using the *GOstats* package.

```
library("org.Gg.eg.db")
library("GOstats")
library("GOSemSim")
paraGO <- new("GOHyperGParams", geneIds=my.geneID3[,2], universeGeneIds=univ.geneID3[,2], 
              annotation="org.Gg.eg.db", ontology="BP", pvalueCutoff=0.05, 
              conditional=TRUE, testDirection="over")
```

GO enrichment analysis for **BP**

```
BP <- hyperGTest(paraGO)
summary(BP)[,c(1,2,7)] # 109
```

```
##         GOBPID       Pvalue
## 1   GO:0051893 0.0005051324
## 2   GO:1901888 0.0005051324
## 3   GO:0002092 0.0018768234
## 4   GO:0072273 0.0018768234
## 5   GO:0007045 0.0022162536
## 6   GO:0072077 0.0036927123
## 7   GO:0034332 0.0037062867
## 8   GO:0045807 0.0060547770
## 9   GO:0090184 0.0060547770
## 10  GO:0010810 0.0068780676
## 11  GO:0061061 0.0080357348
## 12  GO:0007160 0.0082041922
## 13  GO:0048259 0.0089352211
## 14  GO:0051145 0.0089352211
## 15  GO:0051147 0.0096710216
## 16  GO:0001838 0.0123072565
## 17  GO:0048599 0.0123072565
## 18  GO:0048634 0.0130382162
## 19  GO:1901861 0.0130382162
## 20  GO:0071705 0.0143637030
## 21  GO:0023061 0.0158148709
## 22  GO:0010830 0.0161450728
## 23  GO:0030500 0.0161450728
## 24  GO:0051155 0.0161450728
## 25  GO:0015833 0.0169978114
## 26  GO:0034330 0.0169978114
## 27  GO:1903530 0.0187634040
## 28  GO:0035148 0.0204238057
## 29  GO:0060538 0.0240708694
## 30  GO:0060627 0.0240708694
## 31  GO:0048791 0.0249433107
## 32  GO:0001656 0.0251195079
## 33  GO:0048477 0.0251195079
## 34  GO:0048641 0.0251195079
## 35  GO:0002026 0.0254957507
## 36  GO:0003257 0.0254957507
## 37  GO:0003337 0.0254957507
## 38  GO:0006203 0.0254957507
## 39  GO:0006700 0.0254957507
## 40  GO:0006702 0.0254957507
## 41  GO:0006705 0.0254957507
## 42  GO:0006788 0.0254957507
## 43  GO:0007403 0.0254957507
## 44  GO:0009151 0.0254957507
## 45  GO:0009215 0.0254957507
## 46  GO:0009264 0.0254957507
## 47  GO:0010579 0.0254957507
## 48  GO:0010882 0.0254957507
## 49  GO:0030237 0.0254957507
## 50  GO:0031945 0.0254957507
## 51  GO:0031946 0.0254957507
## 52  GO:0032148 0.0254957507
## 53  GO:0032239 0.0254957507
## 54  GO:0032344 0.0254957507
## 55  GO:0032349 0.0254957507
## 56  GO:0032353 0.0254957507
## 57  GO:0032470 0.0254957507
## 58  GO:0033015 0.0254957507
## 59  GO:0033292 0.0254957507
## 60  GO:0034633 0.0254957507
## 61  GO:0034650 0.0254957507
## 62  GO:0038030 0.0254957507
## 63  GO:0045822 0.0254957507
## 64  GO:0045948 0.0254957507
## 65  GO:0046061 0.0254957507
## 66  GO:0046084 0.0254957507
## 67  GO:0046386 0.0254957507
## 68  GO:0046833 0.0254957507
## 69  GO:0046851 0.0254957507
## 70  GO:0046864 0.0254957507
## 71  GO:0046886 0.0254957507
## 72  GO:0050921 0.0254957507
## 73  GO:0050926 0.0254957507
## 74  GO:0050930 0.0254957507
## 75  GO:0051187 0.0254957507
## 76  GO:0051458 0.0254957507
## 77  GO:0051973 0.0254957507
## 78  GO:0055119 0.0254957507
## 79  GO:0060126 0.0254957507
## 80  GO:0060129 0.0254957507
## 81  GO:0061369 0.0254957507
## 82  GO:0070296 0.0254957507
## 83  GO:0071376 0.0254957507
## 84  GO:0090191 0.0254957507
## 85  GO:0090291 0.0254957507
## 86  GO:1900086 0.0254957507
## 87  GO:1900155 0.0254957507
## 88  GO:1900158 0.0254957507
## 89  GO:1902811 0.0254957507
## 90  GO:1903233 0.0254957507
## 91  GO:1903515 0.0254957507
## 92  GO:1903611 0.0254957507
## 93  GO:2000019 0.0254957507
## 94  GO:2000066 0.0254957507
## 95  GO:2000225 0.0254957507
## 96  GO:2000852 0.0254957507
## 97  GO:0010717 0.0302091200
## 98  GO:0034754 0.0302091200
## 99  GO:0060675 0.0302091200
## 100 GO:0072078 0.0302091200
## 101 GO:0051641 0.0302884399
## 102 GO:0051493 0.0338616187
## 103 GO:0019932 0.0356704421
## 104 GO:0042440 0.0356704421
## 105 GO:0043112 0.0356704421
## 106 GO:0060993 0.0356704421
## 107 GO:0061326 0.0356704421
## 108 GO:1903651 0.0356704421
## 109 GO:0034654 0.0372961140
## 110 GO:0014031 0.0388975273
## 111 GO:0043547 0.0388975273
## 112 GO:0045844 0.0414821067
## 113 GO:0051336 0.0416897666
## 114 GO:0031326 0.0420860147
## 115 GO:0032879 0.0465892706
## 116 GO:0006836 0.0476235523
## 117 GO:0007623 0.0476235523
## 118 GO:0008585 0.0476235523
## 119 GO:0072009 0.0476235523
## 120 GO:0090090 0.0476235523
## 121 GO:0090288 0.0476235523
## 122 GO:0072521 0.0488073627
## 123 GO:0060231 0.0492782479
##                                                                                                                           Term
## 1                                                                                        regulation of focal adhesion assembly
## 2                                                                                         regulation of cell junction assembly
## 3                                                                              positive regulation of receptor internalization
## 4                                                                                            metanephric nephron morphogenesis
## 5                                                                                    cell-substrate adherens junction assembly
## 6                                                                                                  renal vesicle morphogenesis
## 7                                                                                               adherens junction organization
## 8                                                                                           positive regulation of endocytosis
## 9                                                                                    positive regulation of kidney development
## 10                                                                                       regulation of cell-substrate adhesion
## 11                                                                                                muscle structure development
## 12                                                                                                        cell-matrix adhesion
## 13                                                                                 regulation of receptor-mediated endocytosis
## 14                                                                                          smooth muscle cell differentiation
## 15                                                                                   regulation of muscle cell differentiation
## 16                                                                                         embryonic epithelial tube formation
## 17                                                                                                          oocyte development
## 18                                                                                      regulation of muscle organ development
## 19                                                                                     regulation of muscle tissue development
## 20                                                                                                 nitrogen compound transport
## 21                                                                                                              signal release
## 22                                                                                       regulation of myotube differentiation
## 23                                                                                           regulation of bone mineralization
## 24                                                                 positive regulation of striated muscle cell differentiation
## 25                                                                                                           peptide transport
## 26                                                                                                  cell junction organization
## 27                                                                                             regulation of secretion by cell
## 28                                                                                                              tube formation
## 29                                                                                           skeletal muscle organ development
## 30                                                                                    regulation of vesicle-mediated transport
## 31                                                                        calcium ion-dependent exocytosis of neurotransmitter
## 32                                                                                                     metanephros development
## 33                                                                                                                   oogenesis
## 34                                                                            regulation of skeletal muscle tissue development
## 35                                                                                regulation of the force of heart contraction
## 36  positive regulation of transcription from RNA polymerase II promoter involved in myocardial precursor cell differentiation
## 37                                                  mesenchymal to epithelial transition involved in metanephros morphogenesis
## 38                                                                                                      dGTP catabolic process
## 39                                                                                    C21-steroid hormone biosynthetic process
## 40                                                                                               androgen biosynthetic process
## 41                                                                                      mineralocorticoid biosynthetic process
## 42                                                                                                              heme oxidation
## 43                                                                                               glial cell fate determination
## 44                                                                                purine deoxyribonucleotide metabolic process
## 45                                                                   purine deoxyribonucleoside triphosphate metabolic process
## 46                                                                                       deoxyribonucleotide catabolic process
## 47                  positive regulation of adenylate cyclase activity involved in G-protein coupled receptor signaling pathway
## 48                                                           regulation of cardiac muscle contraction by calcium ion signaling
## 49                                                                                                    female sex determination
## 50                                                                     positive regulation of glucocorticoid metabolic process
## 51                                                                           regulation of glucocorticoid biosynthetic process
## 52                                                                                     activation of protein kinase B activity
## 53                                                                      regulation of nucleobase-containing compound transport
## 54                                                                                 regulation of aldosterone metabolic process
## 55                                                                     positive regulation of aldosterone biosynthetic process
## 56                                                                         negative regulation of hormone biosynthetic process
## 57                                                      positive regulation of endoplasmic reticulum calcium ion concentration
## 58                                                                                              tetrapyrrole catabolic process
## 59                                                                                                       T-tubule organization
## 60                                                                                                           retinol transport
## 61                                                                                                  cortisol metabolic process
## 62                                                                        non-canonical Wnt signaling pathway via MAPK cascade
## 63                                                                                    negative regulation of heart contraction
## 64                                                                             positive regulation of translational initiation
## 65                                                                                                      dATP catabolic process
## 66                                                                                                adenine biosynthetic process
## 67                                                                                     deoxyribose phosphate catabolic process
## 68                                                                              positive regulation of RNA export from nucleus
## 69                                                                                      negative regulation of bone remodeling
## 70                                                                                                        isoprenoid transport
## 71                                                                         positive regulation of hormone biosynthetic process
## 72                                                                                           positive regulation of chemotaxis
## 73                                                                                           regulation of positive chemotaxis
## 74                                                                                            induction of positive chemotaxis
## 75                                                                                                  cofactor catabolic process
## 76                                                                                                     corticotropin secretion
## 77                                                                                  positive regulation of telomerase activity
## 78                                                                                                relaxation of cardiac muscle
## 79                                                                                 somatotropin secreting cell differentiation
## 80                                                                  thyroid-stimulating hormone-secreting cell differentiation
## 81                                                                negative regulation of testicular blood vessel morphogenesis
## 82                                                                                sarcoplasmic reticulum calcium ion transport
## 83                                                               cellular response to corticotropin-releasing hormone stimulus
## 84                                                     negative regulation of branching involved in ureteric bud morphogenesis
## 85                                                                             negative regulation of osteoclast proliferation
## 86                                                                positive regulation of peptidyl-tyrosine autophosphorylation
## 87                                                                             negative regulation of bone trabecula formation
## 88                                                      negative regulation of bone mineralization involved in bone maturation
## 89                                                                positive regulation of skeletal muscle fiber differentiation
## 90                                                          regulation of calcium ion-dependent exocytosis of neurotransmitter
## 91                                                                 calcium ion transport from cytosol to endoplasmic reticulum
## 92                                                                    negative regulation of calcium-dependent ATPase activity
## 93                                                                               negative regulation of male gonad development
## 94                                                                        positive regulation of cortisol biosynthetic process
## 95                                                                    negative regulation of testosterone biosynthetic process
## 96                                                                                      regulation of corticosterone secretion
## 97                                                                          regulation of epithelial to mesenchymal transition
## 98                                                                                          cellular hormone metabolic process
## 99                                                                                                  ureteric bud morphogenesis
## 100                                                                                               nephron tubule morphogenesis
## 101                                                                                                      cellular localization
## 102                                                                                    regulation of cytoskeleton organization
## 103                                                                                        second-messenger-mediated signaling
## 104                                                                                                  pigment metabolic process
## 105                                                                                                 receptor metabolic process
## 106                                                                                                       kidney morphogenesis
## 107                                                                                                   renal tubule development
## 108                                                                               positive regulation of cytoplasmic transport
## 109                                                                        nucleobase-containing compound biosynthetic process
## 110                                                                                               mesenchymal cell development
## 111                                                                                     positive regulation of GTPase activity
## 112                                                                  positive regulation of striated muscle tissue development
## 113                                                                                           regulation of hydrolase activity
## 114                                                                                regulation of cellular biosynthetic process
## 115                                                                                                 regulation of localization
## 116                                                                                                 neurotransmitter transport
## 117                                                                                                           circadian rhythm
## 118                                                                                                   female gonad development
## 119                                                                                             nephron epithelium development
## 120                                                                     negative regulation of canonical Wnt signaling pathway
## 121                                                         negative regulation of cellular response to growth factor stimulus
## 122                                                                               purine-containing compound metabolic process
## 123                                                                                       mesenchymal to epithelial transition
```

```
# GO similarity
library(corrplot)
```

```
## Warning: package 'corrplot' was built under R version 3.2.5
```

```
goListBP <- summary(BP)[,c(1)]
goSimMatBP <- mgoSim(goListBP, goListBP, ont="BP", measure="Jiang", organism="chicken", combine=NULL)
goSimMatBP <- goSimMatBP[-66, -66]
corrplot(goSimMatBP, is.corr = FALSE, type="lower", tl.col = "black", tl.cex = 0.8)
```

GO enrichment analysis for **MF**

```
ontology(paraGO) <- "MF"
MF <- hyperGTest(paraGO)
summary(MF)[,c(1,2,7)] # 18
```

```
##        GOMFID       Pvalue
## 1  GO:0017124 5.625388e-05
## 2  GO:0030276 5.625388e-05
## 3  GO:0044548 5.763240e-03
## 4  GO:0046872 1.452494e-02
## 5  GO:0003714 1.538956e-02
## 6  GO:0005488 1.853059e-02
## 7  GO:0004112 2.489110e-02
## 8  GO:0004392 2.489110e-02
## 9  GO:0004638 2.489110e-02
## 10 GO:0004639 2.489110e-02
## 11 GO:0004687 2.489110e-02
## 12 GO:0008832 2.489110e-02
## 13 GO:0015056 2.489110e-02
## 14 GO:0032554 2.489110e-02
## 15 GO:0032567 2.489110e-02
## 16 GO:0034632 2.489110e-02
## 17 GO:0043023 2.489110e-02
## 18 GO:0043184 2.489110e-02
## 19 GO:0045159 2.489110e-02
## 20 GO:0047555 2.489110e-02
## 21 GO:0005165 4.917775e-02
## 22 GO:0015020 4.917775e-02
## 23 GO:0030297 4.917775e-02
## 24 GO:0030553 4.917775e-02
## 25 GO:0042056 4.917775e-02
## 26 GO:0048018 4.917775e-02
## 27 GO:0048039 4.917775e-02
##                                                                 Term
## 1                                                 SH3 domain binding
## 2                                                   clathrin binding
## 3                                               S100 protein binding
## 4                                                  metal ion binding
## 5                                 transcription corepressor activity
## 6                                                            binding
## 7                       cyclic-nucleotide phosphodiesterase activity
## 8                              heme oxygenase (decyclizing) activity
## 9                  phosphoribosylaminoimidazole carboxylase activity
## 10  phosphoribosylaminoimidazolesuccinocarboxamide synthase activity
## 11                                myosin light chain kinase activity
## 12                                                  dGTPase activity
## 13                 corticotrophin-releasing factor receptor activity
## 14                                purine deoxyribonucleotide binding
## 15                                                      dGTP binding
## 16                                      retinol transporter activity
## 17                                   ribosomal large subunit binding
## 18             vascular endothelial growth factor receptor 2 binding
## 19                                                 myosin II binding
## 20                       3',5'-cyclic-GMP phosphodiesterase activity
## 21                                     neurotrophin receptor binding
## 22                                  glucuronosyltransferase activity
## 23 transmembrane receptor protein tyrosine kinase activator activity
## 24                                                      cGMP binding
## 25                                          chemoattractant activity
## 26                                         receptor agonist activity
## 27                                                ubiquinone binding
```

```
# GO similarity
goListMF <- summary(MF)[,c(1)]
goSimMatMF <- mgoSim(goListMF, goListMF, ont="MF", measure="Jiang", organism="chicken", combine=NULL)
corrplot(goSimMatMF, is.corr = FALSE, type="lower", tl.col = "black", tl.cex = 0.8)
```

GO enrichment analysis for **CC**

```
ontology(paraGO) <- "CC"
CC <- hyperGTest(paraGO)
summary(CC)[,c(1,2,7)] # 4
```

```
##       GOCCID     Pvalue                                    Term
## 1 GO:0014801 0.02493369     longitudinal sarcoplasmic reticulum
## 2 GO:0042584 0.02493369             chromaffin granule membrane
## 3 GO:0042734 0.02493369                    presynaptic membrane
## 4 GO:0060418 0.02493369                             yolk plasma
## 5 GO:0090534 0.02493369 calcium ion-transporting ATPase complex
## 6 GO:0097470 0.02493369                          ribbon synapse
## 7 GO:0031011 0.04925859                           Ino80 complex
```

```
# GO similarity
goListCC <- summary(CC)[,c(1)]
goSimMatCC <- mgoSim(goListCC, goListCC, ont="CC", measure="Jiang", organism="chicken", combine=NULL)
corrplot(goSimMatCC[-4,-4], is.corr = FALSE, type="lower", tl.col = "black", tl.cex = 0.8)
```

## 4. MeSH enrichment analysis

Then, we perform a MeSH ORA for the category **Chemicals and Drugs** by setting ‘category=“D”’.

```
library(meshr)
library(MeSH.db)
library("MeSH.Gga.eg.db")
meshParams <- new("MeSHHyperGParams", geneIds = my.geneID3[,2], universeGeneIds = univ.geneID3[,2], 
                  annotation = "MeSH.Gga.eg.db", category = "D", database = "gene2pubmed", 
                  pvalueCutoff = 0.05, pAdjust = "none")
meshR <- meshHyperGTest(meshParams)
summary(meshR)[!duplicated(summary(meshR)[,7]),c(1,2,7)] # 63
```

```
##       MESHID      Pvalue                                     MESHTERM
## 2390 D020933 0.001663627                               Neurotrophin 3
## 2802 D056950 0.003665786                    Period Circadian Proteins
## 1078 D005982 0.004311957                      Glutathione Transferase
## 2678 D051499 0.008862701   Receptor, Fibroblast Growth Factor, Type 4
## 2790 D056930 0.008862701                  ARNTL Transcription Factors
## 2808 D064235 0.012202441                            Matrilin Proteins
## 1990 D016203 0.016001235                          CDC2 Protein Kinase
## 2004 D017526 0.016001235                         Receptor, IGF Type 1
## 2021 D018808 0.016001235                    Transcription Factor AP-1
## 2337 D020107 0.016001235                                   Troponin T
## 2712 D055435 0.016001235                                    Myostatin
## 2768 D056926 0.016001235                               CLOCK Proteins
## 7    D002148 0.020233991                  Calmodulin-Binding Proteins
## 2031 D019208 0.020233991            Brain-Derived Neurotrophic Factor
## 2753 D056504 0.020233991                  Chromatin Assembly Factor-1
## 4    D000953 0.025171625                          Antigens, Protozoan
## 5    D001664 0.025171625                                  Biliverdine
## 6    D002122 0.025171625                             Calcium Chloride
## 41   D005395 0.025171625                                    Fish Oils
## 1948 D010908 0.025171625                 Pituitary Hormones, Anterior
## 2020 D018739 0.025171625                     Oncogene Proteins v-erbB
## 2357 D020410 0.025171625   Activated-Leukocyte Cell Adhesion Molecule
## 2365 D020747 0.025171625                     Calcium Channels, T-Type
## 2423 D037241 0.025171625                      Mannose-Binding Lectins
## 2460 D044925 0.025171625 Oxidoreductases Acting on CH-CH Group Donors
## 2664 D050051 0.025171625        Transient Receptor Potential Channels
## 2675 D050863 0.025171625                              Synaptotagmin I
## 2695 D053779 0.025171625             Latent TGF-beta Binding Proteins
## 2710 D054507 0.025171625                  Receptors, Phospholipase A2
## 2711 D054677 0.025171625 Cyclic Nucleotide Phosphodiesterases, Type 1
## 2066 D020033 0.026778865                             Protein Isoforms
## 42   D005819 0.029738908                              Genetic Markers
## 1949 D011972 0.029905815                            Receptor, Insulin
## 1970 D015222 0.029905815                              Sodium Channels
## 2461 D049452 0.031102079                   Green Fluorescent Proteins
## 1316 D007328 0.032538124                                      Insulin
## 1134 D006023 0.033241254                                Glycoproteins
## 2369 D020932 0.041035920                          Nerve Growth Factor
## 1876 D009479 0.044032856                                Neuropeptides
## 2424 D044767 0.045796226                    Ubiquitin-Protein Ligases
## 1437 D008565 0.045977714                            Membrane Proteins
## 1    D000582 0.049711394               Amidophosphoribosyltransferase
## 39   D002262 0.049711394                               Carboxy-Lyases
## 1433 D007329 0.049711394                          Insulin Antagonists
## 1435 D008043 0.049711394                                  Linseed Oil
## 1872 D008747 0.049711394                              Methylcellulose
## 1874 D009251 0.049711394             NADPH-Ferrihemoprotein Reductase
## 1945 D010453 0.049711394                            Peptide Synthases
## 1967 D015081 0.049711394                              2-Naphthylamine
## 1988 D015240 0.049711394                     Phorbol 12,13-Dibutyrate
## 2002 D017493 0.049711394                               Antigens, CD45
## 2017 D018028 0.049711394                       Receptors, Neurotensin
## 2367 D020848 0.049711394                                   Chimerin 1
## 2412 D024502 0.049711394                             alpha-Tocopherol
## 2414 D024745 0.049711394                        Smooth Muscle Myosins
## 2417 D027341 0.049711394          Excitatory Amino Acid Transporter 1
## 2419 D036121 0.049711394                              Receptor, EphA3
## 2665 D050559 0.049711394           beta-Carotene 15,15'-Monooxygenase
## 2685 D051547 0.049711394                             Heme Oxygenase-1
## 2688 D051959 0.049711394 Hu Paraneoplastic Encephalomyelitis Antigens
## 2693 D053613 0.049711394                               Janus Kinase 1
## 2697 D054411 0.049711394                            Receptors, Leptin
## 2707 D054419 0.049711394                       Receptors, Adiponectin
## 2751 D055767 0.049711394                         Immobilized Proteins
## 2806 D062367 0.049711394                         ortho-Aminobenzoates
```

```
# MeSH similarity
library("MeSHSim")
headingListD <- summary(meshR)[!duplicated(summary(meshR)[,7]),c(7)]
meshSimMatD <- mheadingSim(headingListD, headingListD, method="JC")
rownames(meshSimMatD) <- colnames(meshSimMatD) <- summary(meshR)[!duplicated(summary(meshR)[,7]),c(1)]
indexD <- which(meshSimMatD > 0.15 & meshSimMatD != 1, arr.ind = TRUE)
corrplot(meshSimMatD[unique(rownames(meshSimMatD)[indexD[,1]]), 
                     unique(rownames(meshSimMatD)[indexD[,1]])], is.corr = FALSE, type="lower", 
         tl.col = "black", tl.cex = 0.8)
```

Switching to a different category is easily done by the ‘category<-’ function. Here, we use **Diseases** (category = “C”).

```
category(meshParams) <- "C"
meshR <- meshHyperGTest(meshParams)
summary(meshR)[!duplicated(summary(meshR)[,7]),c(1,2,7)] # 12
```

```
##     MESHID      Pvalue                  MESHTERM
## 10 D006528 0.001863934 Carcinoma, Hepatocellular
## 1  D001715 0.025171625             Bird Diseases
## 6  D003327 0.025171625          Coronary Disease
## 7  D003645 0.025171625             Death, Sudden
## 15 D007333 0.025171625        Insulin Resistance
## 32 D010049 0.025171625          Ovarian Diseases
## 33 D013217 0.025171625                Starvation
## 18 D009136 0.029905815      Muscular Dystrophies
## 8  D004370 0.049711394 Duane Retraction Syndrome
## 13 D006956 0.049711394                 Hyperopia
## 30 D009374 0.049711394   Neoplasms, Experimental
## 35 D063646 0.049711394            Carcinogenesis
```

```
# MeSH similarity
headingListC <- summary(meshR)[!duplicated(summary(meshR)[,7]),c(7)]
meshSimMatC <- mheadingSim(headingListC, headingListC, method="JC")
rownames(meshSimMatC) <- colnames(meshSimMatC) <- summary(meshR)[!duplicated(summary(meshR)[,7]),c(1)]
indexC <- which(meshSimMatC > 0.025 & meshSimMatC != 1, arr.ind = TRUE)
corrplot(meshSimMatC, is.corr = FALSE, type="lower", tl.col = "black", tl.cex = 0.8)
```

MeSH ORA for **Anatomy** (category = “A”).

```
category(meshParams) <- "A"
meshR <- meshHyperGTest(meshParams)
summary(meshR)[!duplicated(summary(meshR)[,7]),c(1,2,7)] # 27
```

```
##       MESHID       Pvalue                         MESHTERM
## 2    D002478 2.066345e-05                  Cells, Cultured
## 1199 D007903 2.989189e-04        Lens Capsule, Crystalline
## 1518 D010521 6.318558e-04                       Periosteum
## 1408 D009475 4.381019e-03                Neurons, Afferent
## 1552 D014276 6.008107e-03                 Trigeminal Nerve
## 1638 D017949 1.502469e-02 Retinal Cone Photoreceptor Cells
## 1666 D017950 1.600124e-02                 Ganglia, Sensory
## 1024 D003599 1.971117e-02                     Cytoskeleton
## 1187 D007596 2.023399e-02                           Joints
## 1    D000010 2.517162e-02                   Abducens Nerve
## 1023 D002529 2.517162e-02                Cerebellar Nuclei
## 1111 D006413 2.517162e-02             Hematopoietic System
## 1447 D009847 2.517162e-02                  Olivary Nucleus
## 1558 D014327 2.517162e-02                     Trophoblasts
## 1522 D013687 2.678796e-02                    Telencephalon
## 1575 D016501 2.809079e-02                         Neurites
## 1561 D015672 2.990582e-02        Erythroid Precursor Cells
## 938  D002490 3.139094e-02           Central Nervous System
## 1207 D009432 4.454088e-02                     Neural Crest
## 1678 D020897 4.709480e-02            Organizers, Embryonic
## 1449 D009865 4.935090e-02                          Oocytes
## 1112 D006614 4.946814e-02                         Hindlimb
## 1021 D002525 4.971139e-02                Cerebellar Cortex
## 1197 D007685 4.971139e-02       Kidney Tubules, Collecting
## 1445 D009802 4.971139e-02                 Oculomotor Nerve
## 1520 D012540 4.971139e-02                          Scapula
## 1559 D014928 4.971139e-02                   Wolffian Ducts
## 1676 D019581 4.971139e-02                         Neuropil
```

```
headingListA <- summary(meshR)[!duplicated(summary(meshR)[,7]),c(7)]
meshSimMatA <- mheadingSim(headingListA, headingListA, method="JC")
rownames(meshSimMatA) <- colnames(meshSimMatA) <- summary(meshR)[!duplicated(summary(meshR)[,7]),c(1)]
indexA <- which(meshSimMatA > 0.15 & meshSimMatA != 1, arr.ind = TRUE)
corrplot(meshSimMatA, is.corr = FALSE, type="lower", tl.col = "black", tl.cex = 0.8)
```

MeSH ORA for **Phenomena and Processes** (category = “G”).

```
category(meshParams) <- "G"
meshR <- meshHyperGTest(meshParams)
summary(meshR)[!duplicated(summary(meshR)[,7]),c(1,2,7)] # 22
```

```
##       MESHID      Pvalue                            MESHTERM
## 3513 D016764 0.001130317                       Cell Polarity
## 2961 D014162 0.001826469                        Transfection
## 815  D002455 0.003074289                       Cell Division
## 2841 D013091 0.004682665                     Spermatogenesis
## 941  D004789 0.005589435                   Enzyme Activation
## 3622 D024721 0.016001235                      E-Box Elements
## 3489 D016385 0.017078715                            TATA Box
## 2857 D013379 0.023522569               Substrate Specificity
## 2321 D007333 0.025171625                  Insulin Resistance
## 2840 D009747 0.025171625 Nutritional Physiological Phenomena
## 1096 D005819 0.029738908                     Genetic Markers
## 3609 D020449 0.029905815    Repetitive Sequences, Amino Acid
## 2132 D005822 0.036687202                     Genetic Vectors
## 3549 D020125 0.038877808                  Mutation, Missense
## 2324 D008027 0.042265490                               Light
## 22   D002454 0.046348140                Cell Differentiation
## 1    D001683 0.047094796                   Biological Clocks
## 2364 D009154 0.049287108                            Mutation
## 2221 D006824 0.049711394              Hybridization, Genetic
## 2319 D007112 0.049711394       Immunity, Maternally-Acquired
## 3487 D016009 0.049711394             Chi-Square Distribution
## 3630 D058453 0.049711394                          Emmetropia
```

```
headingListG <- summary(meshR)[!duplicated(summary(meshR)[,7]),c(7)]
meshSimMatG <- mheadingSim(headingListG, headingListG, method="JC")
rownames(meshSimMatG) <- colnames(meshSimMatG) <- summary(meshR)[!duplicated(summary(meshR)[,7]),c(1)]
indexG <- which(meshSimMatG > 0.1 & meshSimMatG != 1, arr.ind = TRUE)
corrplot(meshSimMatG, is.corr = FALSE, type="lower", tl.col = "black", tl.cex = 0.8)
```

## 5. Session Information

```
sessionInfo()
```

```
## R version 3.2.4 (2016-03-10)
## Platform: x86_64-apple-darwin13.4.0 (64-bit)
## Running under: OS X 10.10.5 (Yosemite)
## 
## locale:
## [1] en_US.UTF-8/en_US.UTF-8/en_US.UTF-8/C/en_US.UTF-8/en_US.UTF-8
## 
## attached base packages:
##  [1] grid      parallel  stats4    stats     graphics  grDevices utils    
##  [8] datasets  methods   base     
## 
## other attached packages:
##  [1] MeSHSim_1.2.0            MeSH.Gga.eg.db_1.5.0    
##  [3] meshr_1.6.2              MeSH.Syn.eg.db_1.5.0    
##  [5] MeSH.Bsu.168.eg.db_1.5.0 MeSH.Aca.eg.db_1.5.0    
##  [7] MeSH.Hsa.eg.db_1.5.0     MeSH.PCR.db_1.5.0       
##  [9] MeSH.AOR.db_1.5.0        MeSH.db_1.5.0           
## [11] MeSHDbi_1.6.0            org.Hs.eg.db_3.2.3      
## [13] cummeRbund_2.12.1        Gviz_1.14.7             
## [15] rtracklayer_1.30.4       GenomicRanges_1.22.4    
## [17] GenomeInfoDb_1.6.3       fastcluster_1.1.20      
## [19] reshape2_1.4.1           ggplot2_2.1.0           
## [21] fdrtool_1.2.15           corrplot_0.77           
## [23] GOSemSim_1.28.2          GOstats_2.36.0          
## [25] graph_1.48.0             Category_2.36.0         
## [27] GO.db_3.2.2              Matrix_1.2-6            
## [29] org.Gg.eg.db_3.2.3       RSQLite_1.0.0           
## [31] DBI_0.3.1                AnnotationDbi_1.32.3    
## [33] IRanges_2.4.8            S4Vectors_0.8.11        
## [35] Biobase_2.30.0           BiocGenerics_0.16.1     
## [37] biomaRt_2.26.1          
## 
## loaded via a namespace (and not attached):
##  [1] bitops_1.0-6               matrixStats_0.50.2        
##  [3] RColorBrewer_1.1-2         tools_3.2.4               
##  [5] rpart_4.1-10               Hmisc_3.17-3              
##  [7] colorspace_1.2-6           nnet_7.3-12               
##  [9] gridExtra_2.2.1            formatR_1.3               
## [11] scales_0.4.0               genefilter_1.52.1         
## [13] RBGL_1.46.0                stringr_1.0.0             
## [15] digest_0.6.9               Rsamtools_1.22.0          
## [17] foreign_0.8-66             rmarkdown_0.9.5           
## [19] AnnotationForge_1.12.2     XVector_0.10.0            
## [21] dichromat_2.0-0            htmltools_0.3.5           
## [23] BSgenome_1.38.0            BiocParallel_1.4.3        
## [25] acepack_1.3-3.3            VariantAnnotation_1.16.4  
## [27] RCurl_1.95-4.8             magrittr_1.5              
## [29] Formula_1.2-1              futile.logger_1.4.1       
## [31] Rcpp_0.12.4                munsell_0.4.3             
## [33] stringi_1.0-1              yaml_2.1.13               
## [35] SummarizedExperiment_1.0.2 zlibbioc_1.16.0           
## [37] plyr_1.8.3                 lattice_0.20-33           
## [39] Biostrings_2.38.4          splines_3.2.4             
## [41] GenomicFeatures_1.22.13    annotate_1.48.0           
## [43] knitr_1.12.3               futile.options_1.0.0      
## [45] XML_3.98-1.4               evaluate_0.8.3            
## [47] biovizBase_1.18.0          latticeExtra_0.6-28       
## [49] lambda.r_1.1.7             gtable_0.2.0              
## [51] xtable_1.8-2               survival_2.39-2           
## [53] GenomicAlignments_1.6.3    cluster_2.0.4             
## [55] GSEABase_1.32.0
```
