# Supplementary material for: MeSH-Informed Enrichment Analysis and MeSH-Guided Semantic Similarity Among Functional Terms and Gene Products in Chicken
Source: G3 (Bethesda). 2016 Jun 2;6(8):2447–53. doi: 10.1534/g3.116.031096 (PMC4978898; doi:10.1534/g3.116.031096)
Supplement: Supplemental Material [file supp_g3.116.031096_FileS4.zip › FileS4.html]

Gene Semantic Similarity (Selective sweep data)


# Gene Semantic Similarity (Selective sweep data)

## 1. GO-based Gene Semantic Similarity

```
# compute GO-based semantic similarity between genes identified from BP
library("org.Gg.eg.db")
library("GOSemSim")
library(corrplot)
load("mygeneID3.Rd")
geneSimBP <- mgeneSim(genes=c(my.geneID3[,2]), ont="BP", organism="chicken", measure="Jiang",verbose=FALSE, drop=NULL) # 45
symbolBP <- select(org.Gg.eg.db, rownames(geneSimBP), column="SYMBOL", keytype="ENTREZID")
rownames(geneSimBP) <- colnames(geneSimBP) <- symbolBP[,2]
corrplot(geneSimBP, is.corr = FALSE, type="lower", tl.col = "black", tl.cex = 0.8)
```

```
# compute GO-based semantic similarity between genes identified from MF
geneSimMF <- mgeneSim(genes=c(my.geneID3[,2]), ont="MF", organism="chicken", measure="Jiang",verbose=FALSE, drop=NULL) # 40
symbolMF <- select(org.Gg.eg.db, rownames(geneSimMF), column="SYMBOL", keytype="ENTREZID")
rownames(geneSimMF) <- colnames(geneSimMF) <- symbolMF[,2]
corrplot(geneSimMF, is.corr = FALSE, type="lower", tl.col = "black", tl.cex = 0.8)
```

```
# compute GO-based semantic similarity between genes identified from CC
geneSimCC <- mgeneSim(genes=c(my.geneID3[,2]), ont="CC", organism="chicken", measure="Jiang",verbose=FALSE, drop=NULL) # 46
symbolCC <- select(org.Gg.eg.db, rownames(geneSimCC), column="SYMBOL", keytype="ENTREZID")
rownames(geneSimCC) <- colnames(geneSimCC) <- symbolCC[,2]
corrplot(geneSimCC, is.corr = FALSE, type="lower", tl.col = "black", tl.cex = 0.8)
```

## 2.1. MeSH-based Gene Semantic Similarity

```
# BP
# compute MeSH-based semantic similarity between genes identified from BP
library(MeSH.db)
library("MeSH.Gga.eg.db")
library("MeSHSim")
geneSimMatBP <- matrix(0, ncol=ncol(geneSimBP), nrow=ncol(geneSimBP))
diag(geneSimMatBP) <- 1
n.genes <- ncol(geneSimBP)
for (i in 1:(n.genes-1) ){
  for (j in (i+1):n.genes){
    cat("now i = ", i, " j = ", j, '\n')
g1 <- select(MeSH.Gga.eg.db, colnames(geneSimBP)[i], columns= c("GENEID", "MESHID"), keytype = "GENEID") 
g2 <- select(MeSH.Gga.eg.db, colnames(geneSimBP)[j], columns= c("GENEID", "MESHID"), keytype = "GENEID") 
gList1 <- select(MeSH.db, g1[,2], columns= c("MESHTERM"), keytype = "MESHID") 
gList2 <- select(MeSH.db, g2[,2], columns= c("MESHTERM"), keytype = "MESHID") 
geneSimMatBP[i,j] <- headingSetSim(gList1, gList2, method="JC") 
geneSimMatBP[j,i] <- geneSimMatBP[i,j] 

  }
}
save(geneSimMatBP, file="geneSimMatBP.Rd")

# MF
# compute MeSH-based semantic similarity between genes identified from MF
geneSimMatMF <- matrix(0, ncol=ncol(geneSimMF), nrow=ncol(geneSimMF))
diag(geneSimMatMF) <- 1
n.genes <- ncol(geneSimMF)
for (i in 1:(n.genes-1) ){
  for (j in (i+1):n.genes){
    cat("now i = ", i, " j = ", j, '\n')
g1 <- select(MeSH.Gga.eg.db, colnames(geneSimMF)[i], columns= c("GENEID", "MESHID"), keytype = "GENEID") 
g2 <- select(MeSH.Gga.eg.db, colnames(geneSimMF)[j], columns= c("GENEID", "MESHID"), keytype = "GENEID") 
gList1 <- select(MeSH.db, g1[,2], columns= c("MESHTERM"), keytype = "MESHID") 
gList2 <- select(MeSH.db, g2[,2], columns= c("MESHTERM"), keytype = "MESHID") 
geneSimMatMF[i,j] <- headingSetSim(gList1, gList2, method="JC") 
geneSimMatMF[j,i] <- geneSimMatMF[i,j] 

  }
}
save(geneSimMatMF, file="geneSimMatMF.Rd")

# CC
# compute MeSH-based semantic similarity between genes identified from CC
geneSimMatCC <- matrix(0, ncol=ncol(geneSimCC), nrow=ncol(geneSimCC))
diag(geneSimMatCC) <- 1
n.genes <- ncol(geneSimCC)
for (i in 1:(n.genes-1) ){
  for (j in (i+1):n.genes){
    cat("now i = ", i, " j = ", j, '\n')
g1 <- select(MeSH.Gga.eg.db, colnames(geneSimCC)[i], columns= c("GENEID", "MESHID"), keytype = "GENEID") 
g2 <- select(MeSH.Gga.eg.db, colnames(geneSimCC)[j], columns= c("GENEID", "MESHID"), keytype = "GENEID") 
gList1 <- select(MeSH.db, g1[,2], columns= c("MESHTERM"), keytype = "MESHID") 
gList2 <- select(MeSH.db, g2[,2], columns= c("MESHTERM"), keytype = "MESHID") 
geneSimMatCC[i,j] <- headingSetSim(gList1, gList2, method="JC") 
geneSimMatCC[j,i] <- geneSimMatCC[i,j] 

  }
}
save(geneSimMatCC, file="geneSimMatCC.Rd")
```

## 2.2. MeSH-based Gene Semantic Similarity

```
# BP
load("geneSimMatBP.Rd") # 45
rownames(geneSimMatBP) <- colnames(geneSimMatBP) <- colnames(geneSimBP)
corrplot(geneSimMatBP, is.corr = FALSE, type="lower", tl.col = "black", tl.cex = 0.8, cl.lim=c(0,1))
```

```
# MF
load("geneSimMatMF.Rd") # 40
rownames(geneSimMatMF) <- colnames(geneSimMatMF) <- colnames(geneSimMF)
corrplot(geneSimMatMF, is.corr = FALSE, type="lower", tl.col = "black", tl.cex = 0.8, cl.lim=c(0,1))
```

```
# CC
load("geneSimMatCC.Rd") # 46
rownames(geneSimMatCC) <- colnames(geneSimMatCC) <- colnames(geneSimCC)
corrplot(geneSimMatCC, is.corr = FALSE, type="lower", tl.col = "black", tl.cex = 0.8, cl.lim=c(0,1))
```

## 3. MeSH-based Gene Semantic Similarity

Top five genes under epistatic selection (Beissinger et al. 2015)

```
# find MeSH IDs
library(MeSH.db)
library("MeSH.Gga.eg.db")
library("MeSHSim")
# find entrez gene IDs
g1 <- select(org.Gg.eg.db, "ADCY5", columns= c("ENTREZID"), keytype = "SYMBOL") 
g2 <- select(org.Gg.eg.db, "MYLK", columns= c("ENTREZID"), keytype = "SYMBOL") 
g3 <- select(org.Gg.eg.db, "CAB39", columns= c("ENTREZID"), keytype = "SYMBOL") 
g4 <- select(org.Gg.eg.db, "PIK3CB", columns= c("ENTREZID"), keytype = "SYMBOL") 
g5 <- select(org.Gg.eg.db, "IL1RAP", columns= c("ENTREZID"), keytype = "SYMBOL") 

m1 <- select(MeSH.Gga.eg.db, g1[,2], columns= c("GENEID", "MESHID"), keytype = "GENEID")
gList1 <- select(MeSH.db, m1[,2], columns= c("MESHTERM"), keytype = "MESHID") 
m2 <- select(MeSH.Gga.eg.db, g2[,2], columns= c("GENEID", "MESHID"), keytype = "GENEID")
gList2 <- select(MeSH.db, m2[,2], columns= c("MESHTERM"), keytype = "MESHID") 
m3 <- select(MeSH.Gga.eg.db, g3[,2], columns= c("GENEID", "MESHID"), keytype = "GENEID")
gList3 <- select(MeSH.db, m3[,2], columns= c("MESHTERM"), keytype = "MESHID") 
m4 <- select(MeSH.Gga.eg.db, g4[,2], columns= c("GENEID", "MESHID"), keytype = "GENEID")
gList4 <- select(MeSH.db, m4[,2], columns= c("MESHTERM"), keytype = "MESHID") 
m5 <- select(MeSH.Gga.eg.db, g5[,2], columns= c("GENEID", "MESHID"), keytype = "GENEID")
gList5 <- select(MeSH.db, m5[,2], columns= c("MESHTERM"), keytype = "MESHID") 

# compute MeSH-based semantic similarity between top 5 genes
geneSimMeSH5 <- matrix(0, ncol=3, nrow=3)
rownames(geneSimMeSH5) <- colnames(geneSimMeSH5) <- c("ADCY5", "MYLK", "PIK3CB")
diag(geneSimMeSH5) <- 1
# ADCY5 vs. MYLK
geneSimMeSH5[1,2] <- geneSimMeSH5[2,1] <- headingSetSim(gList1, gList2, method="JC") # JC= 0.2114467
# ADCY5 vs. PIK3CB
geneSimMeSH5[1,3] <- geneSimMeSH5[3,1] <- headingSetSim(gList1, gList4, method="JC") # JC= 0.2178855
# MYLK vs. PIK3CB
geneSimMeSH5[2,3] <- geneSimMeSH5[3,2] <- headingSetSim(gList2, gList4, method="JC") # JC= 0.2896136
corrplot(geneSimMeSH5, is.corr = FALSE,cl.lim=c(0.1,1.0), type="lower", tl.col = "black", tl.cex = 0.8)
```
